# An efficacy and safety report based on randomized controlled single-blinded multi-centre clinical trial of ZingiVir-H, a novel herbo-mineral formulation designed as an add-on therapy in adult patients with mild to moderate COVID-19

**DOI:** 10.1371/journal.pone.0276773

**Published:** 2022-12-06

**Authors:** Shan Sasidharan, Hareendran Nair J., Srinivasakumar K. P., Jerin Paul, Madhu Kumar R., Kannan Rajendran, Anita Ajit Saibannavar, Sonali Nirali

**Affiliations:** 1 Department of Research and Development, Pankajakasthuri Herbal Research Foundation, Pankajakasthuri Ayurveda Medical College Campus, Thiruvananthapuram, Kerala, India; 2 Institute of Biology and Clinical Research (IBCR), Thiruvananthapuram, Kerala, India; 3 Department of Statistics, Vimala College (Autonomous), Thrissur, Kerala, India; 4 Mysore Medical College and Research Institute, Mysuru, Karnataka, India; 5 Saveetha Medical College & Hospital, Chennai, India; 6 RCSM Medical College & CPR Hospital, Dasara Chowk, Kolhapur District, Maharashtra, India; 7 Life Point Multi Specialty Hospital, Pune, Maharashtra, India; Lee Kong Chian School of Medicine, SINGAPORE

## Abstract

**Objective:**

Coronaviruses, hence named because of the crown-like spikes on the viral envelope, are members of Coronaviridae family and Order Nidovirales. SARS-CoV-2 is the seventh human pathogenic coronavirus identified after HCoV-229E, HCoV-OC43, SARS-CoV (SARS-CoV-1), HCoV-NL63, CoV-HKU1, and MERS-CoV. SARS-Cov-2 is highly similar to SARS-CoV. COVID-19 is the corresponding acute disease caused by SARS-CoV-2 that was initially reported in Wuhan, China towards the end of 2019 and spread to millions of humans globally. Unfortunately, limited studies were available on the efficacy of antiviral drugs to treat COVID-19 at the time of this study. ZingiVir-H is an Ayurvedic formulation for use in early therapy of viral disease. This clinical trial was planned to investigate (1) the efficacy and safety of ZingiVir-H and (2) the efficacy of ZingiVir-H as an add-on therapy to the standard of care in hospitalized adults diagnosed with COVID-19.

**Methods:**

A total of 123 eligible subjects as per inclusion criteria were randomized within the study. Three subjects later declined to participate in the study and four subjects didn’t meet inclusion criteria, which brought the final evaluable subject count to 116 for the efficacy and safety endpoint analysis. Thus, a total of 116 patients were equally randomised into two groups, namely, ZingiVir-H and Placebo for this clinical trial. The study patients were assigned to receive either ZingiVir-H or Placebo along with the standard of care as per the National Indian COVID-19 treatment protocol. The time interval until a negative RT-PCR obtained, was evaluated during treatment with ZingiVir-H or Placebo for ten days. Liver and kidney function tests were regularly assessed to ensure the safety profile of ZingiVir-H.

**Results:**

The study found that patients who were administered ZingiVir-H had a median recovery time of 5 days (95% confidence interval (CI) 5–5) when compared to 6 days (95% CI 5–6) in those who received Placebo. Besides, in Ordinal Scale analysis of all the patients treated with ZingiVir-H demonstrated significant redistribution to a better clinical status from ordinal scale 5 to 6 and 7 within five to seven days when compared to that of placebo treatment. The time required for clinical improvement and the number of days needed for hospitalization was significantly less in the ZingiVir-H treated group when compared to placebo. The absence of liver and kidney function changes affirmed the safety profile of ZingiVir-H. No serious adverse events were reported in ZingiVir-H treated patients.

**Conclusion:**

We found that ZingiVir-H is effective and safe in managing COVID-19 infections and delaying the disease progression from mild to moderate and moderate to severe. To the best of our knowledge, this is the first clinical trial report on the efficacy/safety of a herbo-mineral Ayurvedic drug against COVID-19 as of yet.

**Trial registration:**

Clinical Trial Registry of India CTRI/2020/04/024883. Registered on 28/04/2020.

## Introduction

Coronaviruses are single stranded (ss) ribonucleic acid-(RNA) encoded viruses having 65–125 nm diameter and an RNA genome size between 26 and 32 kilobases (kbs). Overall, coronaviruses are categorized into alpha, beta, gamma, and delta groups. The SARS-CoV and MERS are both corona viruses that harm the immune system by causing multisystem inflammatory over-activation and may cause acute respiratory distress syndrome leading to pulmonary failure, ultimately resulting in fatalities. Various parts of the virus are shown in S1 Fig. The Novel Coronavirus Infectious Disease (COVID-19) is a viral respiratory disease caused by a newly identified Severe Acute Respiratory Syndrome Coronavirus 2 (SARS-CoV-2) first reported in Wuhan city of Hubei province in China that killed over 1800 and infected more than 70,000 people within the first 50 days. On 31^st^ December 2019, the local office of WHO alerted its headquarters in New York about “a sudden surge in pneumonia of unknown etiology cases in Wuhan.” The virus quickly received traction and was named Wuhan-Hu-1, an amalgamation of Wuhan Human Influenza Type 1 (Wuhan-Hu-1) based on the then understanding of the virus mimicking symptoms of influenza and the location of origin. However, some Chinese researchers feared racial discrimination. The virus was renamed 2019-nCoV on 7^th^ January 2020 and finally to SARS-Cov-2 on 22^nd^ January 2020 by the Coronavirus Study Group and the International Committee on Taxonomy of Viruses (ICTV) and the disease was named COVID-19 by the WHO. As of 30^th^ January 2020, there had been 7,736 cases that were confirmed and 12,167 cases with strong suspicion of infection that had been reported in China and a total of 82 confirmed cases had been detected in 18 other countries. Hence, WHO declared the SARS-CoV-2 outbreak as a Public Health Emergency of International Concern (PHEIC) on that evening [1]. The virus first travelled from China to Europe (Italy). At that point, there were two known variants of the virus, a less virulent one mainly local to Wuhan and a more virulent one that mainly mutated in Italy and Spain. The European strain entered the USA, mainly via New York and California airports. Parallelly, the cases from Europe, mainly from Italy, entered India. During the Chinese New Year around January, Chinese citizens from Europe visited China and brought viral strains with them during that time. Simultaneously, the virus also spread to the rest of Southeast Asia. The virus was transported to Nepal, Pakistan, the Middle East, and Africa supposedly e.g. via people working on the Chinese Belt and Road project on a different branch. As of February 19, 2022, 10:30 AM EST, there have been 421,565,993 confirmed cases of COVID-19 globally, including 5,873,162 deaths. In case of India, the first virus case was reported on 30^th^ January 2020 in Kerala state. Despite the second-highest population globally, India managed disease spread quite well, implementing timely and strict lockdowns. The total number of cases was limited to a mere 20,000 on 21^st^ April 2020 at the end of the first lockdown period. India had since given up most of its strict measures in favor of economic revival. India had a total of 697,413 cases as of 7^th^ July 2020. However, despite its dense population, India had reported only 18,695 deaths. However, there had been widespread openings throughout the country around that time, resulting in a sudden case number increase with a total of 1,337,024 cases in the country by July 25, 2020. Mortality rate has remained unchanged, raising the total number of deaths at 31,358 in the country, which is a huge jump given its otherwise earlier successes. India (in second place, where America stands first) reported 42,780,917 confirmed cases, including 510,912 deaths, according to the WHO [2].

COVID-19 is a human-to-human transmissible (HHT) potentially lethal viral disease caused by a new coronavirus strain (Novel coronavirus) that differs considerably in genomic structure from all previously identified coronaviruses in the SARS spectrum [3]. This newly identified virus is the seventh in its category which can severely infect humans. SARS-CoV-2 shares a direct lineage with beta coronaviruses emerging from bats and belongs in order Nodovirales and genus Betacoronavirus in the subgenus Sarbecovirus. It shares genomic 96.3% similarity with a purported bat strain reported from Wuhan, (RaTG13), 89% with ZXC21 similar to SARS virus, and 82% nucleotide similarity with SARS-CoV, corroborating to an eventual zoonotic origin [4–6]. But the origin of SARS-CoV-2 is still controversial. Genomic analyses show SARS-CoV-2 likely to be chimeric, most of its sequence closest to bat CoV RaTG13, whereas its receptor binding domain (RBD) on the antigenically most important cell entry and fusogenic protein, the Spike (or S-protein) is almost identical to that of a pangolin CoV [7] and binds with exceptional affinity to its receptor, the human angiotensin-convertase II enzyme (hACE2) on the outside of human cell membranes. Due to these reasons, SARS-CoV-2 may be the outcome of genetic manipulation of an originally not human-specific bat coronavirus intended to promote transmissibility to humans. Both the amino-acid cleavage site at the S-protein and the specific structure of the RBD could result from site-directed mutagenesis, a procedure that does not leave a trace [8]. Furthermore, a recent research work done by Ambati et al [9] also supported the involvement of laboratory associated origin of SARS-CoV-2 by identifying a number of homologies with HIV surface glycoprotein motifs among others, on the Spike. Although not too much promoted in global media, there is indeed ample scientific doubt and now direct evidence about highly unusual properties of SARS-CoV-2 genome sequence, which point to intentional human gain-of-function research leading to increased infectivity for humans, a gain in cell-to-cell fusogenicity, and tissue tropism hence, pathology increase [7]. As mentioned earlier by Ambadi et al [9], the authors raise awareness that a 19-nucleotide sequence patented by Moderna in 2016, US patent 9,587,003 filed on Feb. 4, 2016 -SEQ ID11652 is uniquely found in the virus Spike protein among all known proteins in the BLAST database. Furthermore, the probability of this sequence randomly being present in a 30,000-nucleotide viral genome is 3.21 × 10^−11^ and is from a mismatch repair protein codon optimized for humans. Even further, the mouse (i.e. laboratory) origins of Omicron SARS-CoV-2 are also evidenced by research work conducted by Wei et al. [10].

COVID-19 may vary from mild to moderate illness, severe pneumonia or Acute Respiratory Distress Syndrome (ARDS). At worst, this disease may lead to more fatal conditions like sepsis, septic shock, and eventually multi-organ failure. Unfortunately, there is no unique antiviral drug available on the market that can effectively manage COVID-19 thus far [11]. However, a few vaccinations against COVID-19 infection are currently available [12, 13]. Unfortunately, there is no effective vaccination against infection as of yet. Vaccine efficiency against death has been reported by different scientific papers and studies, with a meagre margin of benefit in a waning mode. However, articles published in reputed journals and the high amount of data analyzed in preprints, carry the message that vaccines do shed similar numbers of virions as unvaccinated individuals, and mRNA Spike exposure actually promotes infection (based on both theoretical Antibody-Dependent Enhancement of Infection and also real-world data), though not symptomatology [14, 15]. This mode of vaccine action is sometimes mentioned in earlier literature as “anti-toxin immunity”. Since there is no specific COVID-19 medication available in clinics, supportive care and on occasion, combination therapy with wide-spectrum antiviral drugs and corticosteroids remains the mainstay of standard clinical treatment to manage this illness [16]. Hence, the world is urged to develop effective therapeutic options in response to the rapid propagation of SARS-CoV-2 [17].

Unlike MERS-CoV, which utilizes the trans-membrane glycoprotein dipeptidyl peptidase 4 for entry into a human cell, SARS-CoV-2 attaches itself to the ACE2 receptor as explained earlier. After a successful entry, the virus releases its genomic material into the host cytoplasm. The RNA is translated to the pp1a and pp1ab polyproteins. This results in the production of proteins that are non-structural at this point and form the replication centre (RTC) within double-membrane vesicles. This in turn, has replication properties and results in the synthesis of a nested set of RNAs that are sub-genomic in nature. Subsequently, these RNAs can then run the code for other proteins continuously that are accessory and structural in nature. The nucleocapsid proteins generated along with the RNA and glycosylated proteins of the viral envelope start coming together to form the virion buds in the transitional Golgi complex and are complete with the fusion of the former with the host membrane and are finally released outside the infected cell to infect other cells. A rapid increase in cytopathy is also observed depending on the viral strain. The cytopathy causes a breakdown in the cellular machinery in various forms such as impacts on the signal pathway of the host cell, issues with cellular functionality, inhibition of genomic activities like interferon gene transcription and translation and increased expression of cytokine/chemokine pathways.

Infection involves the binding of S-glycoprotein trimer RBDs of SARS-CoV-2 to the ACE2 receptor to enter a cell. The other ways of infection include cellular membrane lipid rafts, Neuropilin-1, and cluster of differentiation (CD)-147 as receptors. Genomic analyses show that significant Darwinian selection has caused a convergence of aa-sites in RBD of the S-protein. Coronaviruses generally are (+) strand RNA viruses. These viruses express their RNA-dependent RNA-polymerase enzyme (RdRp) as a genomic complex capable of replication and transcription from a wide-open reading frame (ORF) called ORF1ab. Structural proteins include E, M, N and S proteins expressed via mRNAs. The corresponding genes are the targets for virus detection by RT-PCR tools such as the Corman PCR that targets E-gene and RdRp-gene where E-gene primer is common for bat-related beta coronaviruses the RdRp-gene primer is specific for SARS-CoV-2. The genome of SARS-CoV-2 is over 80% identical to SARS-like bat CoVs and the structural proteins are encoded by S, E, M and N genes, as stated earlier. The orf1ab is the most significant gene that codes for the pp1ab protein and 15 nonstructural proteins (nsp)s. The pp1a protein is encoded by orf1a gene also consists of 10nsps. However, notable differences include the absence of 8a protein and fluctuation in the number of amino acids in 8b and 3c protein in SARS-CoV-2. The 26–32 kb non-segmented ss-RNA genome consists of the 5ʹ-methylated caps and 3ʹ‑polyadenylated tails and is arranged in the order of 5ʹ, replicase genes, genes encoding structural proteins, polyadenylated tail and then the 3ʹ end. ORF1 5ʹ end is partly overlapped by the genome encoding the pp1a and pp1ab replicase polyproteins that in turn, are further cleaved proteases that are basically papain-like cysteine proteases and another protease coded 3CLpro. This results in the generation of non-structural proteins like RdRp and Hel. These act as enzymes further down the line, decrease cellular quality control and interferon activities, and aid in transcription and replication activities.

The 3ʹ 1/3^rd^ of the genome encodes the structural proteins (S, E, M and N). These are involved in receptor binding and virion assembly and are currently the major vaccine and drug design targets. Other non-structural and accessory proteins are also encoded by this region, which may have immunomodulatory effects. This genome structure has been uploaded to NCBI with accession ID NC-045512 as shown in S2 Fig. N proteins of beta coronaviruses share common features in the structure with ordered RNA binding (N1b) and dimerization (N2b) domains separated by short regions with high predicted disorder N1a, N2a, and spacer B/N3 domains. Association of N protein and isolated C-terminal region (domains N2b plus spacer B/N3; residues 210–422) was shown by yeast two-hybrid analysis. Also, purified complete protein was demonstrated to associate into dimers in solution. Viral OLGs have played an important role in the spread of pandemics that allow a single stretch of nucleotide proteins to encode two distinct proteins in different reading frames, leading to point mutations creating resilient varieties capable of spreading the disease at a faster rate. In the case of SARS-CoV-2, ORF3c is an unnamed, unannotated gene which is usually mixed with ORF3b of other SARS-related beta coronaviruses. However, it must be noted that the two genes are not homologous and are located at different genomic sites and reading frames. The former can translate from ribosome profiling and displays immunological properties, whereas the latter does not. A new stop codon (G25563U) is also identified in the gene that has risen to prominence in this current pandemic with multiple recurrences of this mutation. Surprisingly, the newly gained ORF3c stop codon hitchhiked early with haplotype 241U/3037U/14408U/23403G (Spike-D614G), which appeared then to drive the European pandemic spread. The structure of the N2b domain of SARS-CoV and other close viruses showed that the structure of this domain is indeed homo-dimeric and further modelling confirmed that the C-terminal region of this domain mediates self-association into a tetramer, hexamer, and potentially higher oligomeric forms. Co-operative interactions among several interfaces have been highlighted by the self-associating nature of the RNA-binding N1b domain region, showing the possibility of full-length helical filaments. When comparing reference genomes of Neanderthal, Sapiens BUILD34 from 2003, and Sapiens HG38 from 2013, it is clear that each of the 23 human chromosomes can be characterized with evidence of both fractal and resonance periods. These are the differentiating factors for modern humans on a global chromosome-scale, as demonstrated in the phylogenetic tree of coronavirus genomes, as shown in S3 Fig.

Host proteases such as the important, ubiquitous extracellular protease furin, along with its “priming aide” TMPRSS2 enzyme are employed on the S protein for cleavage (S1 and S2 parts are born) to introduce a change in conformation to the latter. This results in an S2 protein hexamer molecular machinery-driven fusion of the virus and host cell membranes. It has been shown that inhibition of TMPRSS blocks SARS-CoV-2 entry and is currently being actively tested to develop drugs against the ailment. It must also be noted that the pattern of expression of ACE2 and TMPRSS2 is specific to cell-types. This means that cRNAseq analyses are fundamental in showing the disease etiology, revealing co-expression of these genes. It must also be noted that TMPRSS2 is more widely expressed than ACE2 which makes hACE2 a principal determinant of susceptibility for infection. scRNAseq has displayed co-expression of ACE2 and TMPRSS2 in pneumocytes in the lungs and goblet secretory cells in the nose. These focal infection points need to be studied further for any cross-cutting relationships. The incubation period of SARS-CoV is 1–4 days, which means the symptoms are visible as soon as a person is infected on mostly the same day. Slightly different incubation period values were also reported (and this wildly varies with strain or variant too) in the literature [18]. This enables the health authorities to manage tracing and quarantine measures effectively. However, the incubation period for SARS-Cov-2 is a whopping 7–14 days, during which the patients are contagious and go on infect unsusceptible people around them. It has been reported that with the D614G variant, each patient can infect 3.77 other people during this period, but further variants produced even a magnitude higher number. This makes tracing the virus extremely difficult and highlights the importance of control measures to reduce the spread, such as handwashing with soap, facial coverings using masks and shields and social distancing.

During drug development directed on receptor binding, entry and pathogenesis, one can note that both SARS-CoV and SARS-CoV-2 use S proteins for broader infections that recognize ACE2 receptors. Several sequence alignments and phylogenetic trees have been generated to identify the specific sequence motifs for attachment required for infection to determine the specificity in S proteins. Two sequence motifs have been identified in the N-terminal domain namely, “MESEFR” and SYLTPG” specific to human SARS CoV-2. Whereas in the RBD, 2 motifs, namely, “VGGNY” and “EIYQAGSTPCNGV” and a disulfide bridge connecting 480C and 488C in the extended loop, have been identified to be the structural determinants for the recognition of the ACE-2 receptor. Genomic analysis of representative coronaviruses from bat, civet and human host sources demonstrated the bat genome (GenBank code: MN996532.1) closest to the SARS-CoV-2 genome with 96.4% of similarity. Recently, CD147, also called emmprin, a trans-membrane glycoprotein, has also been identified as an important possible route for viral entry where upon binding to the mentioned receptors, furin cleaves and TMPRSS2 primes for cleavage the S protein, thus allowing viral entry. Alarmingly, SARS-CoV-2 contains four redundant furin cleavage Pro-Arg-Arg-Ala (PRRA) motifs absent in SARS-CoV or any other betacoronaviridae. This furin cleavage site renders the virus highly multitrophic in organ pathogenesis as well as increases both its human attack rate and pathogenesis. S2 subunit of the S-protein with the RBD, is responsible for binding to ACE2. Thus, these receptors and their sites act as promising targets for drug development.

Multiple antibiotics and antibiotic-combinations have been tried and tested on patients, but no drug has yet been able to prove itself to be a decisive choice. A couple of drugs discussed later in the section on Drug Targets have proven helpful in providing relief and aiding the immune system for rapid recovery. Antivirals such as Hydroxychloroquine, Remdesivir, Itolizumab, Nafamostat, Nitazoxanide, Ribavirin, Penciclovir, Favipiravir, Ritonavir, Baricitinib, AAK1 and Arbidol have been evaluated and exhibited no or very moderately positive results in Phase III clinical trials. Thus, there are currently no antiviral medicines with clinically established efficacy in treating either early, or gravely affected COVID-19 patients [19, 20]. China, where this pandemic originated, has effectively integrated Traditional Chinese Medicine (TCM) and the modern medicine system to treat and manage COVID-19 [21, 22] e.g. with the clinical TCM-based use of flavonoids like hesperidine and naringenin. Likely, Ayurveda, one of the traditional medicinal systems of India could also play a vital role in the management of COVID-19 [23]. From the Ayurvedic perspective, COVID-19 is a contagious fever that can be understood as similar to the sanskrit-worded notion of *Janapadodhwamsavikara* (an epidemic disease) described in one of the ancient basic books of Ayurvedic literature, *Charaka Samhitha*: *Vimana sthana* [24]. Its rapid rate of transmission and route of infection as well as clinical features like cough, dyspnea, anosmia, vomiting and headache are strikingly similar to those of the contagious fevers described in *Sushruta Samhita* [25].

The Ministry of AYUSH (*Ayurveda*, Yoga and Naturopathy, Unani, Siddha & Homeopathy) in India has been proactive in mitigating the COVID-19 crisis right from the beginning. They released a health advisory that includes simple, household measures to enhance our immunity [26]. Other AYUSH stakeholders, such as educational institutes and the pharmaceutical sector, have also undertaken various clinical studies for COVID-19 prevention or management because of the pandemic’s scale and the urgency for effective therapies. These efforts undoubtedly increased the number of AYUSH clinical studies on COVID-19. The most frequent interventions investigated in AYUSH trials were Arsenicum Album, Ashwagandha, AYUSH-64, and Guduchi Ghan Vati [27].

Considering this scenario, our study targeted to test a new herbo-mineral formulation called ZingiVir-H in COVID-19 cases. ZingiVir-H recorded significant in-vitro antiviral activity when tested against SARS-CoV-2 strains [28]. A previous ZingiVir-H pilot study recorded its significant antiviral and antipyretic effect when administered in viral fever patients [29]. Two different clinical toxicity studies on ZingiVir-H and its ingredients revealed that the drug is safe and free from any toxic effects [30, 31]. The ingredients used for the formulation of ZingiVir-H are provided in Table 1. *Zingiber officinale*, one of the key ingredients used for this formulation and its phytochemicals have prominent antiviral activity [31]. The major phytochemicals found in *Zingiber officinale* are 6-Shogoals, 6-Gingerol, and Allicin were reported to have significant antiviral effects by preventing viral attachment and internalization. 6-Gingerol was also reported to have significant binding affinity and interaction with multiple targets of COVID-19, including viral proteases, RNA binding protein and spike protein [32]. Quercetin, a common flavonoid phytochemical found in ZingiVir-H ingredients such as *Cyperus rotondus*, *Zingiber officinale*, and *Hedyotis corymbosa* was also reported to have an antiviral effect in COVID-19 [32] and in influenza by interacting with viral HA (hemagglutinin) protein and thereby inhibiting virus entry into cells. Quercetin may additionally allow for dissection of the viral life cycle and has potential therapeutic use to reduce the virus production in the body with very low associated toxicity [33]. Another ingredient, *Syzygium aromaticum*, has been shown to have antiviral activities against the Herpes simplex virus, as well as anti-inflammatory and antipyretic characteristics due to the presence of an active phytochemical called Eugenin [34]. *Syzygium aromaticum* is renowned in Ayurveda as a strong analgesic medicine because of its high flavonoid content. *Syzygium aromaticum* is also an effective expectorant and has antioxidant, hepatoprotective, immunomodulatory and antistress properties. It also has medicinal properties such as the potential to treat diarrhea, nausea, vomiting, and headaches. Mercurid, one of the ingredients used to formulate ZingiVir-H, was also reported to have antiviral properties. Mercurid was primarily employed to combat avian influenza, respiratory syncytial virus, adenovirus, rotavirus, and other viruses [35].

**Table 1:** Composition of ZingiVir-H tablets.

SL. No	Common/Vernacular name	Scientific/Botanical name	Form used	Quantity/ 500 mg
**1**	Lavanga /Grampoo/Clove	*Eugenia caryophyllus*	Dried clove bud powder	55 mg
**2**	Adraka /Inchi/Ginger	*Zingiber officinale*	Fresh ginger rhizome juice and aqueous extract	200 mg
**3**	Mustha/Muthanga	*Cyperus rotundus*	Dried roots and rhizomes powder	35 mg
**4**	Parpata/Parpatakapullu	*Hedyotis corymbosa*	Dried whole plant powder	30 mg
**5**	Ajamoda/Ayamodhakam	*Trachyspermum ammi*	Dried fruit powder	60 mg
**6**	Hingulam/Chayilyam	HgS (Mercuric sulphide)	Purified and processed as per texts.	20 mg
**7**	Haratala/Thaalakam	As_2_S_3_ (Arsenic trisulphide)	Purified and processed as per texts	10 mg
**8**	Pharmaceutical grade starch	Starch	-	90 mg

In this article, we are reporting the results of our study showcasing the efficacy and safety of ZingiVir-H as an add-on therapy in managing mild to moderate COVID-19 infected patients. We hope our study findings will raise awareness in the world as well as in the medical-scientific community regarding the emergence of a promising novel oral herbo-mineral Ayurvedic drug having significant anti-coronaviral activity. Hence, we presume that this formulation might be a powerful asset to humanity if introduced to the mainstream of COVID-19 treatment and its management.

## Materials and methods

The present trial aimed to assess the efficacy and safety of ZingiVir-H as an add-on therapy along with the standard of care in the management of COVID-19 infected adults between 18 and 60 years of age.

### Study drug

The study drug is ZingiVir-H, a herbo-mineral Ayurvedic preparation in tablet form for oral administration. ZingiVir-H (500 mg) tablets are manufactured at a Good Manufacturing Practices (GMP) approved production line at Pankajakasthuri Herbals India Pvt. Ltd. situated at Poovachal, Thiruvananthapuram, Kerala, India.

### Study sites

We enrolled patients at (1) KR Hospital (Mysore Medical College and Research Institute, Mysuru, Karnataka, India), (2) RCSM Medical College & CPR Hospital (Bhausinghaji Road, Dasara Chowk, Kolhapur District, Maharashtra, India), (3) Saveetha Medical College & Hospital, (Saveetha Nagar, Thandalam, Chennai, India) and (4) Life Point Multi-Specialty Hospital (No. 145, Sr, 1, Mumbai Pune Bypass Road, Near Sayaji Hotel, Wakad, Pune, Maharashtra, India). The study was coordinated and the site-specific training was managed by the Institute of Biology and Clinical Research (www.ibcrglobal.org), No: 55, Chirayath Building, Kaimanam, Thiruvananthapuram, Kerala, India. The study protocol and other essential documents have been followed consistently across the four study sites after approval by the respective Institutional Ethics Committees. The details regarding the multi-centric study sites were provided as a S1 Table.

### Study design

This single-blind randomized controlled multi-centre clinical trial [36] was adopted according to the ICH harmonized guideline integrated addendum to ICH E6 (R1): Guidelines for Good Clinical Practice ICH E6 (R2) ICH Consensus Guideline (Current Step 4 version dated November 9, 2016) and strictly adhering to the rules and regulations of ICMR [37] with ethical clearance/approval from all the participating study sites. After ethical approval, the study was registered under the Clinical Trial Registry of India (CTRI) with registration number CTRI/2020/04/024883 dated 28/04/2020. The present protocol was designed as a randomized controlled single-blinded multi-centre clinical trial to investigate the efficacy and safety of ZingiVir-H as an add-on therapy in hospitalized adults diagnosed with mild and moderate COVID-19 infection. The trials were conducted between May 4 and July 16, 2020. The chosen patients were equally divided and assigned to either the ZingiVir-H arm or the Placebo arm. The treatment plan was scheduled in a manner that one tablet (500 mg) was administered orally to the patients every 3±1 hours between 6 AM and 9 PM in a given day (i.e., 6 AM, 9 AM, 12 Noon, 3 PM, 6 PM & 9 PM) for a minimum period of 10 days to a maximum 15 days, depending on the clinical conditions and disease outcome. Placebo tablets of similar colour, sensory qualities and consistence to ZingiVir-H tablets were distributed in the same timing to the Placebo group patients. Both the study arms received the standard of care as per the COVID-19 management policies advised from time-to-time by the Ministry of Health and Family Welfare, Government of India. Blood samples were withdrawn at regular intervals to analyze kidney and liver functions. The evaluations were performed during baseline (Day-01) and at the end of the study. Clinical laboratory test results and descriptions of any major adverse events reported during the trial period are included in the safety evaluations.

### Sample size calculation

Sample size computation was done on the basis of a comparison of two proportions and based on this power analysis it was decided to at least include 54 samples in each of the groups (arms) (S2 Table).

### Randomization & blinding

The study drug was assigned and orally administered in a single-blind method. At the same time, the trial participants and Legally Acceptable Representatives (LAR) were blinded to the study arm information. This study strictly followed a single-blinding technique to ensure that the participants had no prior knowledge of their group assignment (i.e., allocation concealment). The labeling of the study drug kit was done centrally by the Study Director and Medical Monitor of the study at the sponsor end. Further, the numbered kits were dispatched to all participating sites where the kits numbered between 001 and 099 were ZingiVir-H drug and kits numbered between 101 and 199 were Placebo. The information regarding the number of study drug kits was shared with the investigation team just before the commencement of the study. All the subjects with odd enrollment numbers were assigned with study drug and the even enrollment number subjects were assigned with Placebo. Through this pattern, the study team was also assured that they did not influence the participant randomization. The randomization was carried out based on a subject’s Informed Consent Form (ICF) signing date and time. The sponsors also ensured this through telephonic communication and by getting randomization information immediately after the study drug administration. They have also reassured that the recruitment happened chronologically with the corresponding investigational product assignment. It was further advised to the study team that, in case of a medical emergency where it is imperative to disclose the treatment arm in which a study subject belongs, the investigating team could break the blinding, if they obtained authorization from either the Medical Monitor or the Sponsor. It was also instructed that any broken blinding should be clearly justified and enunciated in the patient notes and the Case Report Form (CRF) [38].

### Inclusion criteria

The following were the main inclusion criteria for this study. Patients of both sexes aged between 18 and 60 years old who were willing and able to give a written Informed Consent Form prior to undergoing study procedures. Patients with peripheral capillary oxygen saturation (SpO_2_) >94% in room air at screening and SARS-CoV-2 infections which were established by Reverse Transcriptase Polymerase Chain Reaction (RT-PCR) test within 5 days before randomization were included in this study. Radiographic evidence of pulmonary infiltrates in the patients was also a prerequisite.

### Exclusion criteria

Subjects who were unable to provide an Informed Consent Form; pregnant or breastfeeding females; patients who have hemostasis impairment because of any recent surgery; history of trauma; major neurological complications; patients who were not able to take oral medications and patients with prolonged QTC-interval in baseline ECG (>500 ms). Likewise, active cancer patients and chronic kidney failure patients were explicitly excluded from this study.

### Withdrawal criteria

The study allows subjects to withdraw from treatment if they experienced unbearable adverse event(s) or serious adverse event(s) or if the study drug administration is contraindicated by the standard of care medications or hypercapnic. The investigation could also be terminated based on a study subject request or at the investigator’s discretion on any clinical aspects.

### Ordinal scale assessment: Baseline to end of the study

An Ordinal scale on the ratio of improvement on a 7-point scale [1. Death; 2. Hospitalized; on invasive mechanical ventilation or Extracorporeal Membrane Oxygenation (ECMO); 3. Hospitalized, on non-invasive ventilation or high flow oxygen supplying devices; 4. Hospitalized, requiring low flow supplemental oxygen; 5. Hospitalized, not requiring supplemental oxygen and require only ongoing medical care (COVID-19 related or otherwise); 6. Hospitalized, not requiring supplemental oxygen-no longer required ongoing standard medical care (other than per protocol ZingiVir-H administration) and 7. Discharge from hospital] between the treatment groups was followed in the study. The Ordinal scale is a technique for assessing the clinical status which was clearly documented daily from Day-01 to End of Treatment (EOT) and further up to the End of Study (EOS). Each day, the worst score from the preceding day was recorded. The Ordinal scale was referred and developed based on the WHO R&D Blueprint of Novel Coronavirus COVID-19 Therapeutic Trial Synopsis. This scoring method was also used in the Adaptive COVID Treatment Trial (ACTT) sponsored by the National Institute of Allergy and Infectious Diseases (NIAID), USA [39].

### Therapeutic intervention

The randomized subjects on the ZingiVir-H arm or Placebo arm were administered orally with one tablet (500 mg) of either study drug or Placebo, each administered every 3±1 hours between 6 AM and 9 PM on a given day (6 AM, 9 AM, 12 Noon, 3 PM, 6 PM, 9 PM) for a minimum duration of 10 days to a maximum of 15 days as per the clinical conditions and improvement of the disease. Further, standard precautions were applied at all times according to risk assessment for all patients, whenever providing any diagnostic and care services. The general medical care and treatment protocols applied to the study subjects were strictly followed based on the clinical management protocol for COVID-19 approved by the Ministry of Health and Family Welfare, Government of India. The symptomatic study subjects were treated with antipyretics for managing fever and pain, antitussives for cough and further ensured a balanced diet and adequate daily fluid intake. Since hydroxychloroquine tablets were prescribed for only one study patient who was in the Placebo group, the efficacy of HCQS didn’t make any impact on our study nor did we find any significance. A few (6) patients with systemic pulmonary infiltrates in the placebo group were given antibiotics (Azithromycin 500 mg twice daily) [40] to manage the condition.

### Dosing visits—day-1 (baseline) to day15 (end of treatment)

The first dosage of study drug (ZingiVir-H) or Placebo was given to all trial participants within 4 hours of their enrollment in the study. The ZingiVir-H was administered orally every 3±1 hours between 6 AM and 9 PM on a given day (6 AM, 9 AM, 12 Noon, 3 PM, 6 PM, 9 PM); i.e., six tablets or 3 gm of ZingiVir-H per day for a minimum period of 10 days to a maximum of 15 days depending on the clinical outcome of the study participants. After the first dose, changes in concomitant drugs, adverse events, substantial changes in vital signs, and respiratory failure occurrences (if any) were meticulously recorded daily until the treatment was completed.

### Study assessments and outcome measures

Before the baseline visit for study enrollment, all study subjects underwent an RT-PCR test to confirm COVID-19. Prior to completing the Informed Consent Form, demographics and medical history (all known information, such as health history/relevant surgeries/interventions) of the study subjects were collected. The symptoms/signs of vitality recorded were body temperature, pulse rate in beats per minute (BPM), blood pressure in millimeters of mercury (mmHg), breaths per minute and any comorbidity symptoms. The following parameters were recorded in a physical examination: general appearance, Head, Eye, Ear, Nose & Throat (HEENT), neck, respiratory, cardiovascular, chest, abdomen, lymphatic, musculoskeletal and extremities, skin, neurologic examination and finally about the concomitants (including medications, fluids and blood products administered within the 7 days before randomization).

### Outcome measures by laboratory investigations

The RT-PCR test for COVID-19 was performed on all study participants, especially on the 5^th^, after the initiation of the study and further on any day based on investigator discretion until the patient becomes RT-PCR negative [41]. The liver and kidney function tests were performed during baseline and end of the study (day 30) to assess the safety during the treatment of ZingiVir-H in the study subjects. The overall intervention period of the study was 30 days. The primary efficacy of the trial outcome was measured as the Odds Ratio for Improvement on a 7-point Ordinal scale [*Ref*. *Section*: 2.8] on Day 15 [Time Frame: Day 15 from the day of study inclusion]. The Odds Ratio compared the treatment groups’ chances of improving on the Ordinal scale (ZingiVir-H *vs*. Placebo). The outcome measures also included the assessment of body temperature of participants in the ZingiVir-H arm and Placebo arm on Days 1, 3 and 5 of the clinical trial.

The study also measured safety outcomes on any changes between the treatment groups regarding pulmonary infiltrates, adverse events or any significant increase in laboratory values.

### Data collection and analysis

The information gathered and documented in the source notes were correctly transcribed into the study-specific Case Report Forms. The Case Report Forms were in compliance with the protocol, evaluation criteria, and study procedures.

The primary sponsorship and monetary/material support were provided by Pankajakasthuri Herbal Research Foundation, Pankajakasthuri Ayurveda Medical College Campus, Kattakada, Thiruvananthapuram, Kerala, India.

### Trial registration and ethical statement

The study was registered under the Clinical Trial Registry of India (CTRI) with registration number CTRI/2020/04/024883. The trial was carried out in strict accordance with the Declaration of Helsinki and its modified recommendations for Good Clinical Practices.

### Statistical analysis of data

The collected data were analysed using different statistical techniques. Descriptive measures were evaluated for the selected variables and some variables are graphically represented using bar diagrams and growth curves. Survival analysis using the Cox proportional hazards model (log-log method for identifying the confidence intervals) with Kaplan-Meier plot was performed to study the significance of ZingiVir-H effects. Wald test was applied on the predictor variables of the Cox model. Further we performed the Log-Rank test. It is a statistical method for comparing the distribution of time until the occurrence of an event of interest in independent groups. It indicates whether survival between two groups is significantly different but does not indicate how different they are and the same is utilized in this work to identify the difference in survival between ZingiVir-H and Placebo [42, 43]. Odds ratio and relative risk with its confidence intervals are calculated to verify the improvement in ordinal scale (ZingiVir-H vs Placebo) [44]. Friedman test was performed to check for the significant difference in temperature of the patients collected at three-time points.

## Results

**Fig 1:**
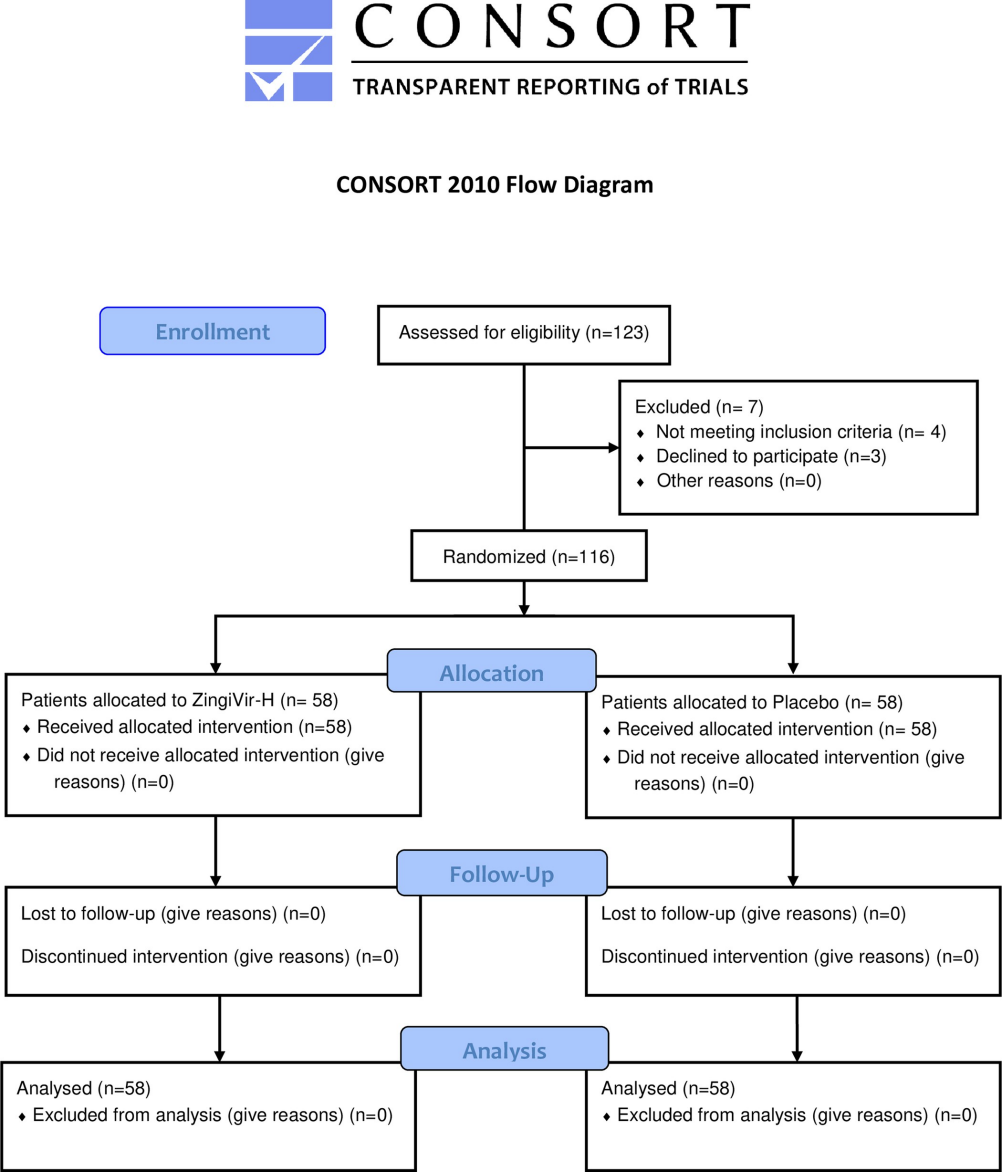
Trial flow chart of the study.

A total of 123 eligible subjects as per inclusion criteria were randomized within the study. However, three subjects withdrew their consent during the treatment period and four subjects left the study site because of medical advice (pre-existing medical conditions), which brought the final evaluable subject count to 116 for the efficacy and safety endpoint analysis. Out of 116 randomized patients, 58 patients were assigned to the ZingiVir-H group and 58 to the Placebo group, as shown in Fig 1. The baseline parameters of the ZingiVir-H and placebo groups are provided in Table 2 and the baseline parameters of the patients are detailed in Table 3. Complying with the hospital policy and the National COVID-19 Treatment Protocol, all the candidates were assigned to receive study drug ZingiVir-H or Placebo along with the standard of care treatment. The primary efficacy examination was done on an intention-to-treat basis with all randomly assigned patients. The time to clinical improvement was calculated after all patients who had tested for RT-PCR negative after the administration of study drug (Either ZingiVir-H (3 g/day) or Placebo) for at least ten days. In this trial, one hundred and one patients (87.06%) were categorized on the Ordinal scale 5. However, such patients had a mild-to-moderate disease and required additional medical care. The remaining 15 subjects (12.94%) were found asymptomatic according to the Ordinal scale 6 and administered only the study drug during the treatment period. The median age of study patients was 35 (Inter Quartile Range (IQR) 27–47). The ZingiVir-H group had a median age of 42 (IQR 30–50), while the Placebo group had 31.5 (IQR 26–44.25).

**Table 2:** The baseline parameters, count of patients in each ordinal scale and the descriptive measures of temperature of ZingiVir-H and Placebo groups.

Variables			Treatment group		
			Placebo	ZingiVir-H	Total
Center	Number of subjects in Center I		26	25	51
	Number of subjects in Center II		20	20	40
	Number of subjects in Center III		7	7	14
	Number of subjects in Center IV		5	6	11
Gender	Female		7	16	23
	Male		51	42	93
Age: Mean ± Standard Deviation			34 ± 11	40 ± 12	37 ± 12
Day 1	Ordinal Scale 5 (Medical care and ZingiVir-H/Placebo)		50 (43.10%)	51 (43.97%)	101 (87.07%)
	Ordinal Scale 6 (ZingiVir-H/Placebo only)		8 (6.9%)	7 (6.03%)	15 (12.03%)
	Ordinal Scale 7 (Discharge)		0	0	0
Day 5	Ordinal Scale 5 (Medical care and ZingiVir-H/Placebo)		27 (23.28%)	13 (11.21%)	40 (34.48%)
	Ordinal Scale 6 (ZingiVir-H/Placebo only)		8 (6.9%)	5 (4.31%)	13 (11.21%)
	Ordinal Scale 7 (Discharge)		23 (19.83%)	40 (34.48%)	63 (54.31%)
Day 7	Ordinal Scale 5 (Medical care and ZingiVir-H/Placebo)		8 (6.9%)	0	8 (6.9%)
	Ordinal Scale 6 (ZingiVir-H/Placebo only)		5 (4.31%)	0	5 (4.31%)
	Ordinal Scale 7 (Discharge)		45 (38.79%)	58 (50%)	103 (88.79%)
Day 13	Ordinal Scale 5 (Medical care and ZingiVir-H/Placebo)		0	0	0
	Ordinal Scale 6 (ZingiVir-H/Placebo only)		0	0	0
	Ordinal Scale 7 (Discharge)		58 (50%)	58 (50%)	116 (100%)
Median recovery time (In days) with 95% CI			5 (5–5)	6 (5–6)	5 (5–6)
Body temperature		Day 1	98.2 ± 0.6	98.4 ± 0.6	98.3 ± 0.6
		Day 2	98.2 ± 0.6	98.3 ± 0.5	98.2 ± 0.4
		Day 3	98.2 ± 0.5	98.2 ± 0.4	98.2 ± 0.4
		*p*-value	0.1554	0.526	0.286

**Table 3:** Baseline characteristics of study subjects.

	ZingiVir-H		Placebo		All patients (n = 116)
	Male	Female	Male	Female	
Sex	48	10	45	13	Male-93 numbers Female-23 numbers
Pulse	77.22±6.24	77.2±2.89	80.34±3.63	80±4.43	78.75±5.09
Weight	60.53±6.56	62.94±9.35	61.34±5.13	62.55±5.28	61.27±6.17
Height	1.63±0.06	1.63±0.06	1.58±0.05	1.58±0.04	1.61±0.06
Oral/Tympanic temperature	37.33±1.28	37.79±0.52	36.71±0.40	36.47±0.54	37.04±0.97
**Number of patients detected abnormalities**					
General appearance	7	3	6	1	17
Skin	1	Nil	1	Nil	2
Eyes, ears, nose & throat	16	4	17	5	42
Cardiovascular	Nil	Nil	1	Nil	1
Respiratory	17	3	10	2	32
Abdomen	1	Nil	Nil	1	2
Lymph nodes	Nil	Nil	Nil	Nil	Nil
Muscular-Skeletal	2	Nil	3	1	6
Neurological	Nil	1	1	Nil	2
diabetes mellitus	1	Nil	1	Nil	2
Hypertension	Nil	Nil	1	Nil	1
Dyslipidemia	Nil	Nil	Nil	1	1
Other extremities	Nil	Nil	Nil	Nil	Nil

**Fig 2:**
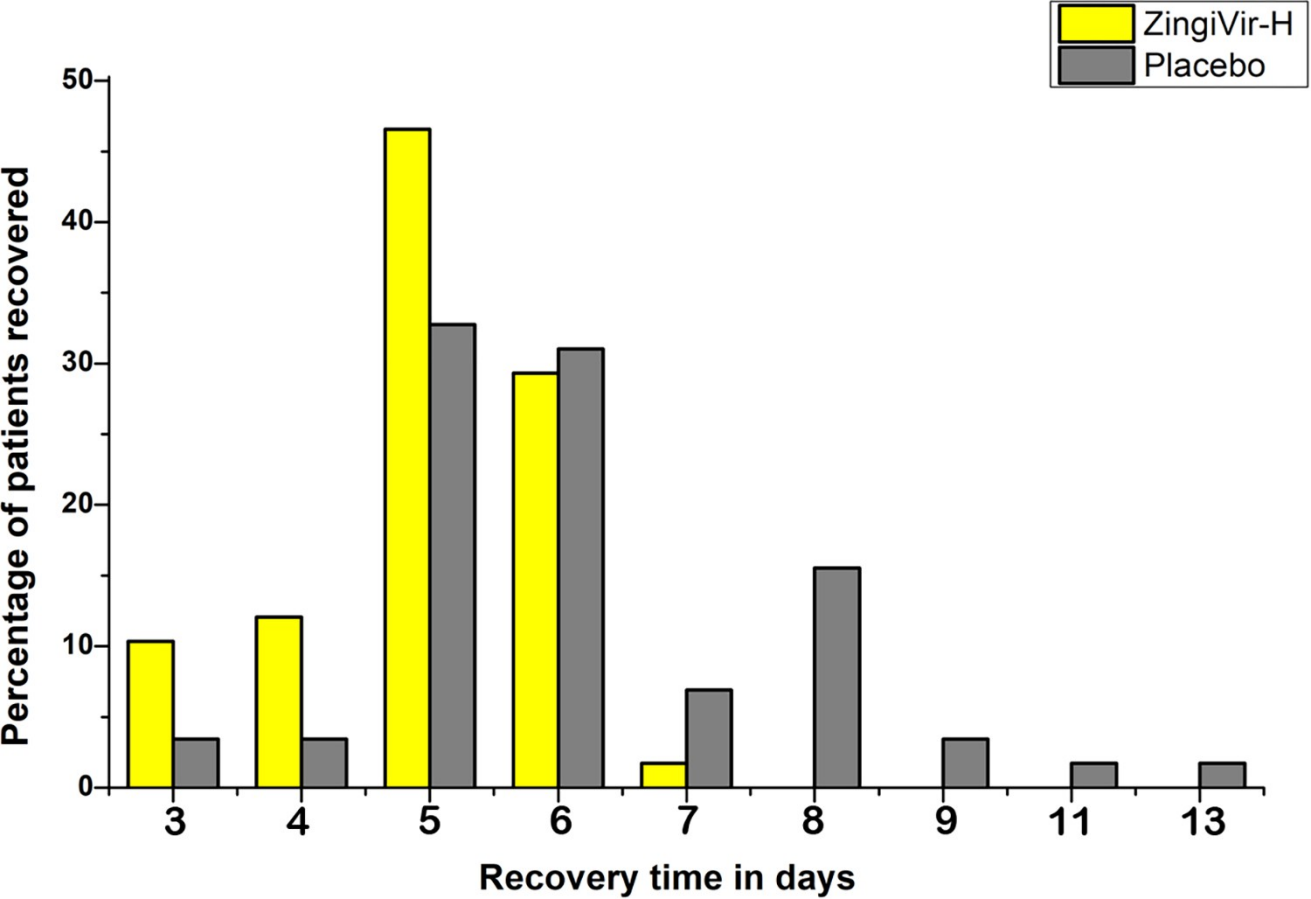
Percentage of patients recovered from COVID-19 infection.

**Fig 3:**
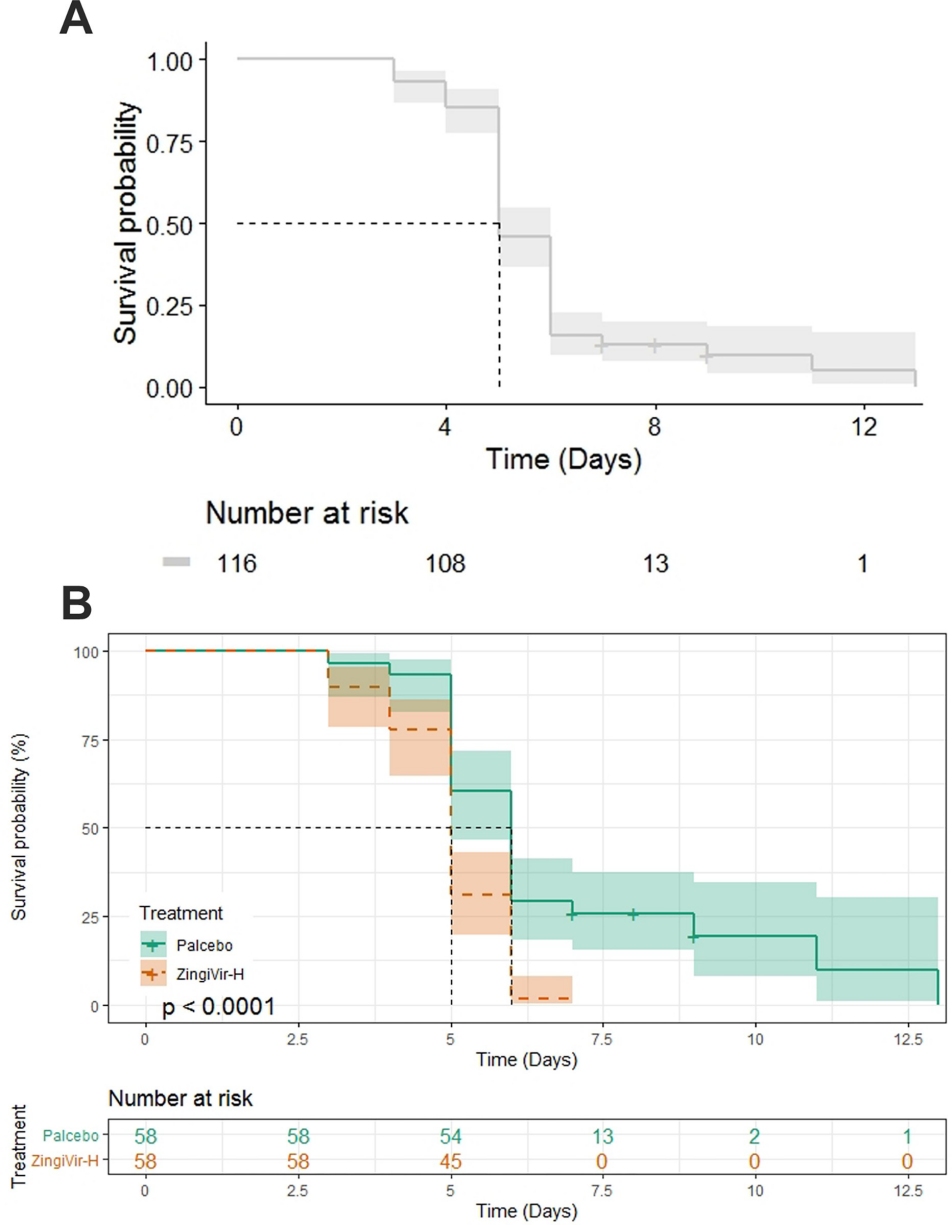
(A) Overall median recovery time. (B) Median recovery time of ZingiVir-H and Placebo treatments.

All of the patients in the ZingiVir-H group recovered within 3 to 6 days during the study. Moreover, the maximum number of patients (27) recovered from COVID-19 according to RT-PCR was recorded on day 5 (Fig 2). On the other hand, patients in the Placebo group have recovered within 4 to 13 days. The maximum number of patients recovered from COVID-19 was on day 5 and 6 where the patient count was 19 and 18, respectively (Fig 2). The patients who received ZingiVir-H in this study had a median recovery time of 5 days (95% CI 5–5) as compared to 6 days (95% CI 5–6) in those who received Placebo (Fig 3). The overall median recovery time was also determined and recorded as 5 days (95% CI 5–6) (Fig 3A). Though the median recovery rate of placebo is 6, some placebo administered patients still took 13 days for disease recovery when compared to the lesser recovery time of all patients (7 days) who were administered the study drug. Hence, it can be concluded that the time required for clinical improvement and the number of days needed for hospitalization was significantly less in the ZingiVir-H treated group when compared to placebo and standard of care. This signifies the role of ZingiVir-H in the effective management of COVID-19. The Log-Rank test was applied to confirm the significance of recovery time between the ZingiVir-H and Placebo groups, and it was recorded that ZingiVir-H had a prominent recovery time improvement with a *p*<0.0001. Hence, the ZingiVir-H-treated group recovered much faster than the Placebo group. Significant results for the ZingiVir-H treated group include a Wald test score of 20.91 with a *p*<0.0001. The hazard ratio in the Cox proportional hazards model is 2.619 (95% CI 1.733–3.956) with a highly significant *p*-value<0.0001, indicated that the ZingiVir-H treated group had a significant recovery period. After inclusion of age in the Cox proportional hazards model the hazard ratio become 2.7520 (95% CI 1.8043–4.198) with a significant *p*-value <0.0001 and p value corresponding to age is obtained as 0.274. The score of the Wald test is 22.1 with *p-*value <0.001 shows significance in survival for ZingiVir-H treatment.

### Evaluation of time to clinical improvement for ordinal scale values 5 and 6

The time to clinical improvement was also evaluated for Ordinal scales values 5 and 6 using the same mode of analysis mentioned above.

**Fig 4:**
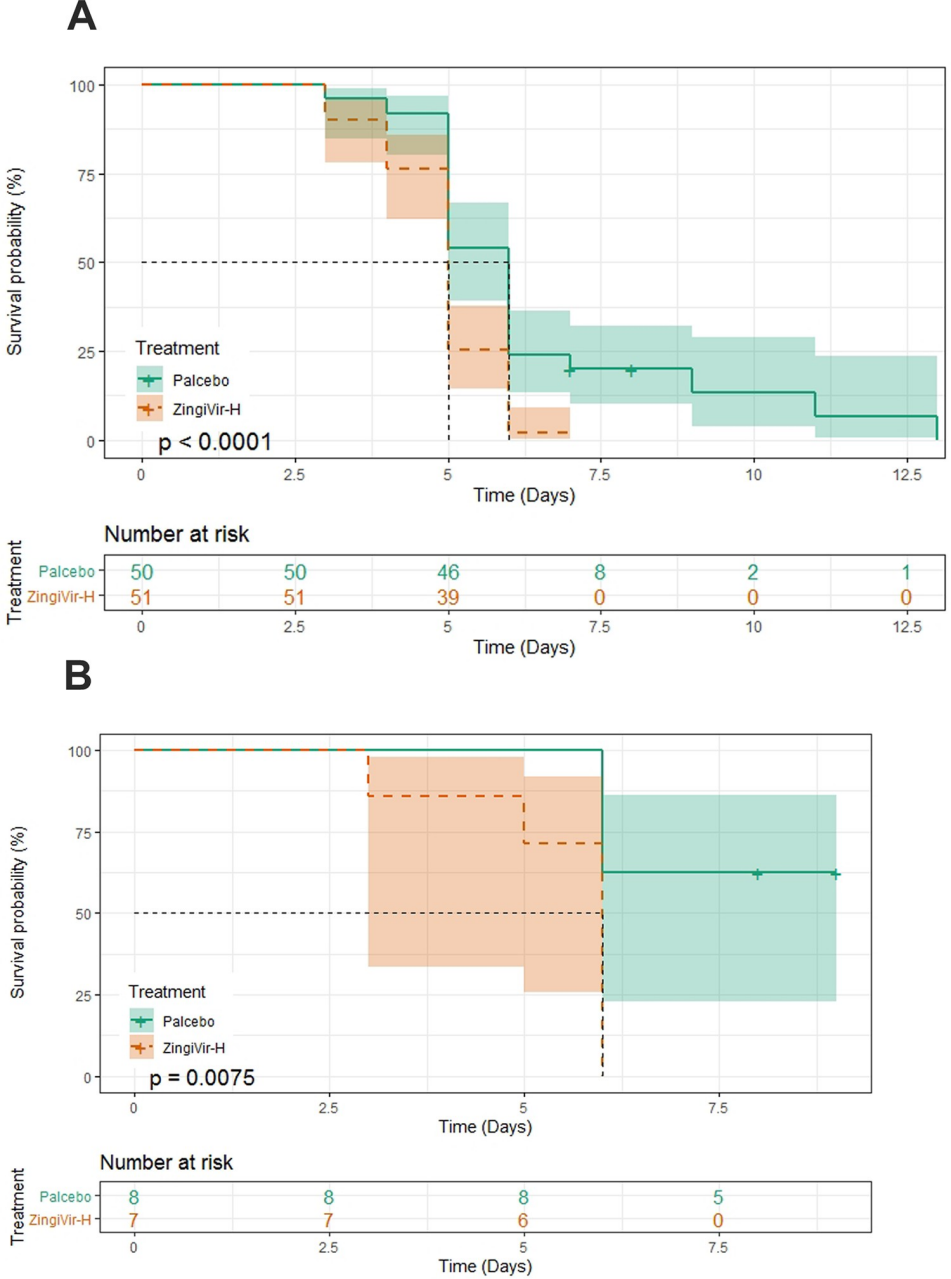
(A) Ordinal Scale-5: Median recovery time of ZingiVir-H and Placebo treatments. (B) Ordinal Scale-6: Median recovery time of ZingiVir-H and Placebo treatments.

Under Ordinal scale 5, the overall median recovery time was observed as 5 days (95% CI 5–5). The median recovery time required for ZingiVir-H received patients was identified as 5 days (95% CI 5–5) and that of Placebo was identified as 6 days (95% CI 5–6) (Fig 4A). The Log-Rank test result with a *p*<0.0001 confirmed the significant difference in the median recovery time. The hazard ratio obtained for this case was 2.356 (95% CI 1.531–3.624).

In Ordinal scale 6, the overall median recovery time was obtained as 6 days (95% Lower CI is 6). Specifically, considering the maximum median recovery time for the ZingiVir-H group was calculated as 6 days and that of the Placebo group was more than 6 days (Fig 4B). The significant difference existing in the median recovery time between the two groups was confirmed with the Log-Rank test results (*p*<0.0001). The hazard ratio obtained in this case was 5.899 (95% CI 1.446–24.07).

From both the scales, it can be concluded that a better recovery time was with the ZingiVir-H group than that of the Placebo group. The Schoenfeld Residual Test was used to validate the PH assumption on both scales (Ordinal scale 5: *p*-value = 0.65, Ordinal scale 6: *p*-value = 0.51).

### Analysis of primary end point by ordinal scale

In the study design, the primary end point was set at day 15 from the day of study inclusion. However, in the trial, all patients recovered by day 13. So, the improvement in the ordinal scale from 5 and 6 to 7 is verified on day 5 instead of day 15. Since most of the patients in the two groups are recovered on the fifth day. The odds ratio calculated on day 5 based on number of patients cured out of the total patients in two groups is 3.3816 (95% CI 1.5725–7.2720) and the corresponding relative risk is 1.7391 (95% CI 1.2117–2.4962). The odds ratio indicated the difference in clinical status toward the ordinal scale 7 for the ZingiVir-H group vs. the placebo group. Furthermore, all the patients treated with ZingiVir-H demonstrated significant change in clinical status from ordinal scale 5 to 6 and 7 within five to seven days when compared to that of placebo (Table 2).

### Analysis of body temperature of the patients

**Fig 5:**
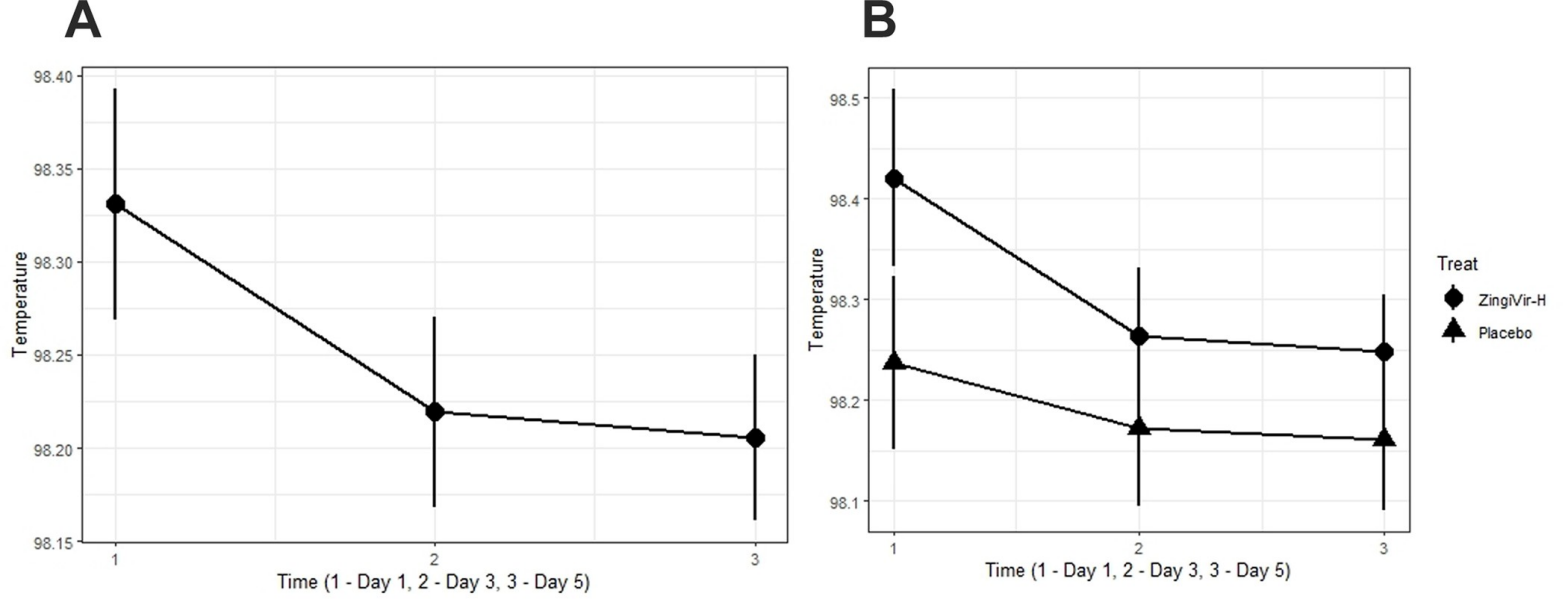
(A) The graph of reduction in the temperature of all patients considered for the trial. (B) The graph of reduction in temperature corresponding to each.

The body temperature of the patients was assessed at three-time points, viz., day 1, day 3, and day 5 of the clinical trial (Fig 5). Friedman’s test was used to verify the difference in temperature data over these time points. The test result for the whole dataset showed that the temperature was not significantly different when evaluated on the time points during the trial (*p*-value = 0.1554). The test was also conducted to compare the temperature over three-time points within each treatment arm. The Friedman’s test for the ZingiVir-H group over three-time periods yielded a *p*-value of 0.286, and the test for the Placebo resulted in 0.526. The test results indicated that even in each group, the temperature difference does not exist.

### Safety analysis of ZingiVir-H before and after the study period

The safety analysis of ZingiVir-H before and after the study period was assessed using the Liver Function Test (LFT) and Renal Function Test (RFT).

LFT and RFT were considered as the benchmark for assessing any damage to the liver and kidney tissue due to the intake of ZingiVir-H in the specific trial dosage. To analyse them both, Friedman’s tests were performed. The results conspicuously demonstrated no significant difference in the mean LFT and RFT values of all the variables between the initial and final time points of intervention. In addition to this, the LFT and RFT values were clinically within the normal range for a healthy person, which indicated no adverse effect or impairment to the liver and kidney functions of the trial volunteers (Table 4). Thus, it was concluded that the ZingiVir-H is not hepatic/renal toxic and is safe to use at the recommended dosage.

**Table 4:** Liver Function Test and Renal Function Test of ZingiVir-H treated group before treatment and after treatment.

Test	Before treatment (Day 1)	After treatment (Day 30)	*P* value
**Liver Function Test**			
AST	35.8± 25.8	30 ±7.65	0.4444
ALT	31.7± 28.9	28.4 ±8.08	0.9999
AST: ALT	1.34 ±0.559	1.08 ±0.263	0.9104
GGPT	43.0 ±62.2	21.2 ±7.12	0.5062
ALP	74.9 ±26.4	74.4 ±10.8	0.1272
Total Bilirubin	0.673± 0.431	0.938 ±0.499	0.128
Direct Bilirubin	0.214 ±0.114	0.178 ±0.107	0.129
Indirect Bilirubin	0.459± 0.355	0.76±0.555	0.2123
Total protein	7.16 ±1.07	7.57 ±0.274	0.1447
Albumin	4.37 ±0.434	4.71 ±0.437	0.6217
AG Ratio	1.52 ±0.292	1.66 ±0.261	0.1447
**Kidney Function Test**			
Urea	23.6± 8.32	21 ±3.28	0.416
Creatinine	0.849 ±0.213	0.858 ±0.206	0.2998
Uric Acid	4.47 ±1.49	4.82 ±1.22	0.1272
Calcium	8.88 ±0.859	9.46 ±0.261	0.2773
Phosphorus	5.60± 3.04	5.86± 3.16	0.2089
Alkaline Phosphorus	74.9±26.4	74.4±10.8	0.1272
Sodium	141 ±2.68	140 ±3.11	0.2714
Potassium	4.73± 0.934	4.61± 0.689	0.5519
Chlorine	100± 3.50	99.9 ± 1.17	0.5885

### Serious adverse effect

No adverse events or serious adverse reactions were reported in patients while performing the trials. A few ZingiVir-H treated patients recorded a rise in electrolyte levels, particularly phosphorous and sodium, which was evaluated by the investigation team and judged clinically non-significant and mentioned in the patient source notes. The study reported six mild adverse reactions, which were not related to the trial drug (Table 5). The events subsided and none of the events were reported as SAE or SAE criteria.

**Table 5:** Adverse reactions noted in the study which were not related to the trial drug.

Sl. No.	Event Name	Severity & IP relatedness	Medication prescribed	Dosage	Outcome
1.	Diarrhea	Mild/ Not related	Racecadotril (Tab)	100mg/BD	Resolved
2.	Headache	Mild/ Not related	Ibuprufen (Tab)	400mg/BD	Resolved
3.	Mouth Ulcer	Mild/ Not related	Choline salicylate (Gel)	1 mg	Resolved
4.	Headache	Mild/ Not related	Diclofenac (Tab)	100mg/BD	Resolved
5.	Diarrhea	Mild/ Not related	Diphenoxylate (Tab)	2.5mg/BD	Resolved
6.	Headache	Mild/ Not related	Paracetamol (Tab)	400mg/BD	Resolved

## Discussion

The analysis of the data from this trial found that ZingiVir-H treatment added to the standard of care was associated with significant clinical improvement and safety outcomes in asymptomatic, mild to moderately affected COVID-19 patients when compared to the Placebo which was associated only with the standard of care. Results from the 116 patients with data available after randomization displayed that patients who administered ZingiVir-H had a median recovery time of 5 days (95 percent confidence interval (CI) 5–5) when compared to 6 days (95 percent CI 5–6) in those who received Placebo. All patients infected with COVID-19 assigned to the ZingiVir-H group recovered within 3 to 7 days during the study. According to RT-PCR, the maximum number of patients (27) recovered from COVID-19 was recorded on day 5. This was nearly 50% of the total patients enrolled in the ZingiVir-H group (58). This means the maximum recovery period of ZingiVir-H concentrated on the 5^th^ day after the initiation of the treatment. On the other hand, patients in the Placebo group recovered within 3 to 13 days. Here the maximum number of patients recovered from COVID-19 was on day 5 and day 6 where the patient count was 19 and 18, respectively. So, it was clear that the days taken by the Placebo to recover from the COVID-19 infection were longer when compared to the ZingiVir-H recovery interval. The number of patients recovered from the infection in the 5^th^ and 6^th^ days covers more than 60% of the total patients in the Placebo group. Furthermore, in Ordinal Scale analysis all the patients treated with ZingiVir-H demonstrated significant betterment of distribution in clinical status from ordinal scale 5 to 6 and 7 within five to seven days when compared to that of placebo. Burman et al. reported that the average recovery period of COVID-19 patients in India is 25 days (95% confidence interval 16.14 days to 33.86 days). Moreover, only 4% of the patients get cured of the infection after 10 days of treatment [45]. Therefore, it is clearly evident that the recovery period in patients who underwent ZingiVir-H administration was far better than the normal recovery rate in India. Our study results demonstrated that ZingiVir-H had a median recovery time of 5 days. Additionally, our findings were comparable to Remdesivir’s results in treating individuals with severe COVID-19, with a numerical reduction of 5 days in median time to clinical improvement [46].

As per the inclusion criteria of our protocol, the study subjects had to be RT-PCR positive for COVID-19. All study subjects recruited in the present clinical trial came into the mild and moderate COVID-19 patient category. Further analysis of body temperature does not establish a criterion for recruitment. Moreover, the study subjects presented had no fever, and had normal body temperature during their admission to this study. Since the patients in both the ZingiVir-H and the Placebo group hadn’t have fever, the antipyretic effect of the study drug couldn’t be demonstrated. But patients who were administered ZingiVir-H had better viral clearance and better recovery time when compared to those who received Placebo, and this result signified the antiviral effect of ZingiVir-H. However, we have demonstrated the antipyretic effect of ZingiVir-H earlier through a pilot study where ZingiVir-H recorded a significant decrease in body temperature in patients admitted with viral fever [29].

The participating candidates in this trial were diverse in terms of disease duration and severity at the time of inclusion. Patient data points were included in the “Per Protocol Assessment Criteria” during the data analysis. The data collected from the study sites based on the Investigational Product dispensing log and compliance log confirmed that the patients were regular with the study medications. The study participants reported no irritation or difficulty in swallowing the study drug. During the first, third, and fifth days of the study, there was no significant difference in body temperatures between the ZingiVir-H and Placebo groups. Moreover, mean baseline body temperature and that after intervention were clinically normal. Fever is also a prevalent symptom in COVID-19 patients who were hospitalized, according to previous studies. The degree of temperature increase may reflect the severity of inflammation in the body [47]. The subjects who participated in this trial reported no mortality or serious adverse events, indicating that the study drug is safe in asymptomatic, mild and moderate COVID-19 infected patients.

### Study limitations

The limitations we faced during this assessment were mainly due to the COVID-19 lockdown protocols implemented in the various study sites across the country. The assessment of LFT and RFT parameters at the end of the study (day 30) were performed only in 79 study patients. Various challenges existed during the trial period such as government-directed restrictions on the movement of people from place to place; keeping essential services only; risk of infection to trial participants and healthcare staff; requirement for self-isolation if the research staff became sick; requirement for restricting movement if close contact with an infected person occurs; and the knock-on effects of staffing changes; limited research staff got training on the study procedures owing to the pandemic situation prevalent at that period; site monitoring visits; the inability of the study patients who left the facility after becoming COVID-19 negative to report the hospital for follow up visits due to quarantine norms etcetera which made deviations from the study protocol on patient visits. Despite all these challenges, the EOS was performed in 68.1% of the study populations.

Furthermore, ZingiVir-H had an excellent safety profile in this trial, but more testing in larger patient cohorts with diverse ethnicities or illness states is needed for additional validation. Nevertheless, this is the first randomized controlled trial of a herbo-mineral medication in individuals with mild to moderate COVID-19 infections as per our knowledge. These findings further suggest additional larger controlled studies to confirm the treatment benefit of ZingiVir-H in severe COVID-19 patients.

## Conclusions

The study drug ZingiVir-H has recorded a recovery time of 5 days as compared to 6 days in those who received Placebo in patients with mild and moderate COVID-19. Upon analysis of the clinical trial data, it was clear that the time required for clinical improvement and the number of days needed for hospitalization was significantly less in the ZingiVir-H treated group when compared to placebo and standard of care. No significant adverse events or adverse medication responses were reported in the ZingiVir-H-treated patients. Based on our results, ZingiVir-H may be effective and safe in managing the COVID-19 infections and delaying the disease progression from mild to moderate and moderate to severe. Finally, we can conclude that the human suffering caused by this fatal pandemic disease cannot be expressed just in numbers, and that each day encapsulates the frustration, dread, and immense loss suffered by millions of human beings all over the globe. We have a conviction that converges with the rollout of new effective vaccines; ZingiVir-H could indeed help to restore the state of life of common people inflicted by this pestilence to normalcy.

## Supporting information

S1 Checklist CONSORT 2010 checklist of information to include when reporting a randomised trial*. (DOC)

S1 Fig SARS-Cov2 and its interactions with the surface proteins of the target cell by Marta Palma Rodríguez (Graduate Student, Hospital General Universitario de Valencia). Submitted to Protein Data Bank CellPAINT contest. Retrieved as per terms of CC BY 4.
(DOCX)

S2 Fig SARS-CoV-2/Wuhan-Hu-1 reference sequence (NC_045512). (DOCX)

S3 Fig Phylogenetic tree of corona virus genomes. Traced by National Institutes of Health (NIH).
(DOCX)

S1 Table Multi-centric study details. (DOCX)

S2 Table Sample size computation for comparison of two proportion. (DOCX)

S1 File (DOCX)

S2 File (DOCX)

S3 File (DOCX)
