# Supplementary material for: An efficacy and safety report based on randomized controlled single-blinded multi-centre clinical trial of ZingiVir-H, a novel herbo-mineral formulation designed as an add-on therapy in adult patients with mild to moderate COVID-19
Source: PLoS One. 2022 Dec 6;17(12):e0276773. doi: 10.1371/journal.pone.0276773 (PMC9725144; doi:10.1371/journal.pone.0276773)
Supplement: S1 Fig — Submitted to Protein Data Bank CellPAINT contest. Retrieved as per terms of CC BY 4. (DOCX) [file pone.0276773.s002.docx]

**Supplemental figure**

**Fig S1. SARS-Cov2 and its interactions with the surface proteins of the target cell by Marta Palma Rodríguez (Graduate Student, Hospital General Universitario de Valencia). Submitted to Protein Data Bank CellPAINT contest. Retrieved as per terms of CC BY 4**

**
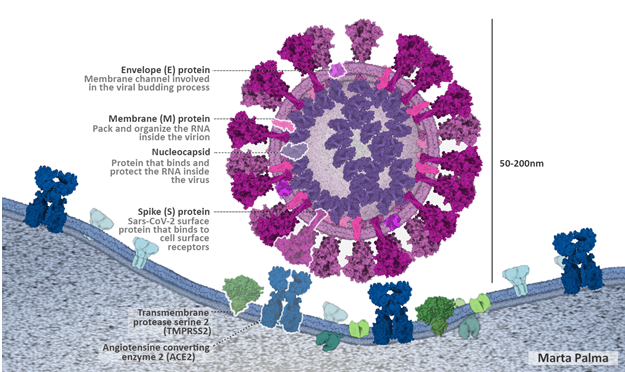
**
