# Supplementary figures and images for: An efficacy and safety report based on randomized controlled single-blinded multi-centre clinical trial of ZingiVir-H, a novel herbo-mineral formulation designed as an add-on therapy in adult patients with mild to moderate COVID-19
Source: PLoS One. 2022 Dec 6;17(12):e0276773. doi: 10.1371/journal.pone.0276773 (PMC9725144; doi:10.1371/journal.pone.0276773)

**Supplemental figure**

**Fig S2. SARS-CoV-2/Wuhan-Hu-1 reference sequence (NC_045512)**

**
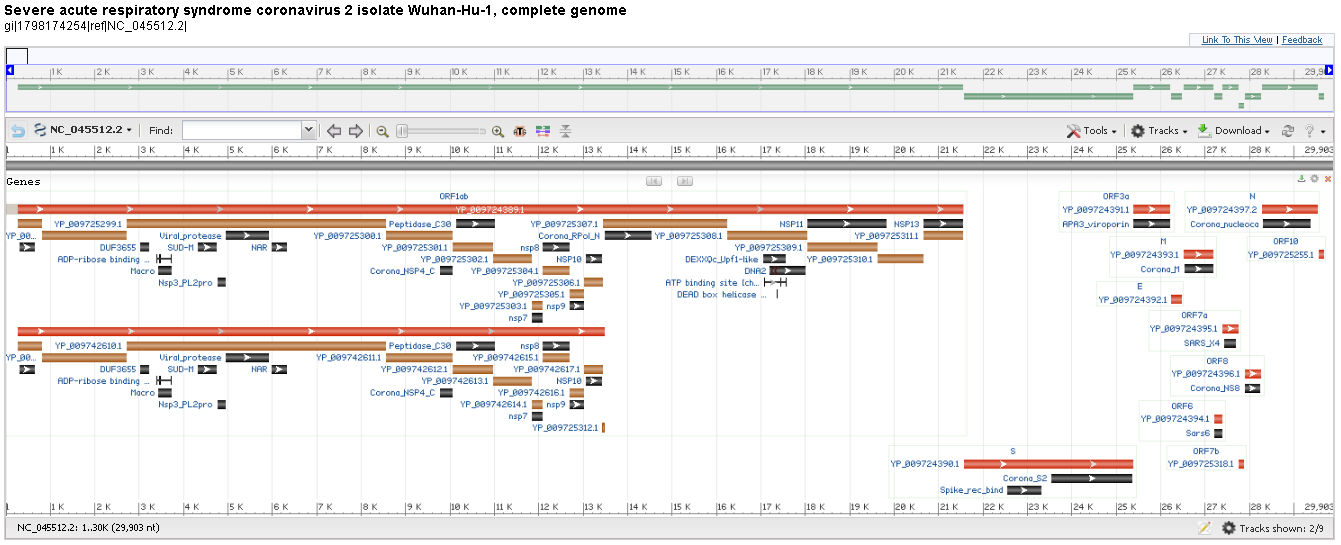
**

Supplement: S2 Fig — (DOCX) [file pone.0276773.s003.docx]
