# Supplementary material for: An efficacy and safety report based on randomized controlled single-blinded multi-centre clinical trial of ZingiVir-H, a novel herbo-mineral formulation designed as an add-on therapy in adult patients with mild to moderate COVID-19
Source: PLoS One. 2022 Dec 6;17(12):e0276773. doi: 10.1371/journal.pone.0276773 (PMC9725144; doi:10.1371/journal.pone.0276773)
Supplement: S3 Fig — Traced by National Institutes of Health (NIH). (DOCX) [file pone.0276773.s004.docx]

**Supplemental figure**

**Fig S3. Phylogenetic tree of corona virus genomes. Traced by National Institutes of Health (NIH)**

**
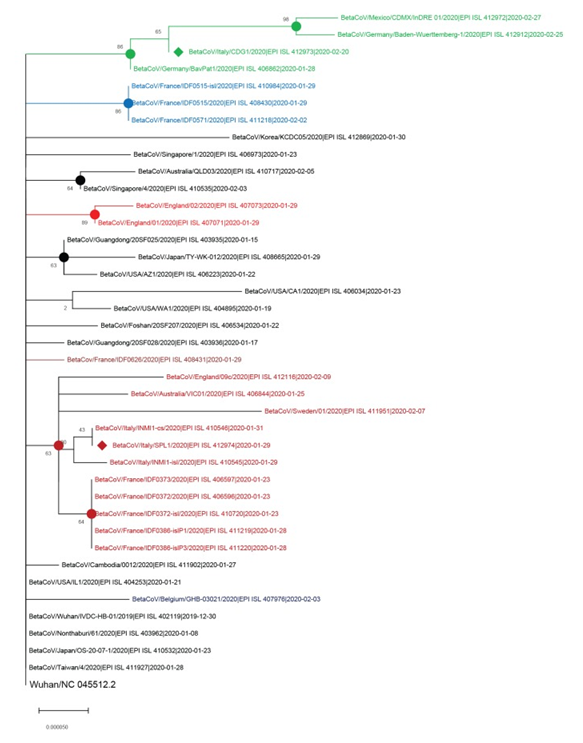
**
