# Supplementary material for: An efficacy and safety report based on randomized controlled single-blinded multi-centre clinical trial of ZingiVir-H, a novel herbo-mineral formulation designed as an add-on therapy in adult patients with mild to moderate COVID-19
Source: PLoS One. 2022 Dec 6;17(12):e0276773. doi: 10.1371/journal.pone.0276773 (PMC9725144; doi:10.1371/journal.pone.0276773)
Supplement: S1 Table — (DOCX) [file pone.0276773.s005.docx]

**Supplementary Table**

**S2 Table: Sample Size computation for comparison of two proportion**

The equation for the calculation of sample size for the comparison of two proportion is given by

$$n=\frac{2 p q {(Z_{1-\frac{\alpha}{2}}+Z_{1-\beta})}^{2}}{{(p_{1}-p_{2})}^{2}}$$

The terms involved in this equation are explained below

In this study we have two groups viz, placebo control and case group.

| Placebo-Control | Placebo-Control | Cases | Cases |
| --- | --- | --- | --- |
| % of Cured (PCR Negative) | % of Non-cured (PCR Positive) | % of Cured (PCR Negative) | % of Non-cured (PCR Positive) |
| p_1_ | q_1_ | p_2_ | q_2_ |

$p=\frac{p_{1}+p_{2}}{2}$ and $q=\frac{q_{1}+q_{2}}{2}$

$Z_{1-\frac{\alpha}{2}}$ is the Z value at ‘α’ level of significance

| **Level of significance** | $Z_{1-\frac{\alpha}{2}}$ **Value** |
| --- | --- |
| 5% | 1.96 |
| 1% | 2.58 |

$Z_{1-\beta}$ is the Z value at (1-β)% power

| **Power** | $Z_{1-\beta}$ **Value** |
| --- | --- |
| 80% | 0.84 |
| 90% | 1.28 |

$p_{1}-p_{2}$ sometimes notated by *d* known as clinically significance difference (Minimum difference (between the groups) which the investigator considers as clinically relevant and it is decided by the investigator)

For this study specifically I have taken the following values

$$p_{1}=0.6, p_{2}=0.85, q_{1}=0.4, q_{2}=0.15, p=0.725, q=0.275, and p_{1}-p_{2}=-0.25$$

$Z_{1-\frac{\alpha}{2}}=1.96$ at ‘5%’ level of significance

$Z_{1-\beta}=0.84$ at 80% power

Now

$$n=\frac{2*0.725*0.275*{(1.96+0.84)}^{2}}{{(-0.25)}^{2}}=48.82641$$

A 10% dropout is expecting, which will be equal to $= 0.1*48.82641 = 4.882641$

Therefor the total sample size $= 48.82641+4.882641=53.70905\approx54$

Approximately 54 samples should be required in each group to detect a clinically significance difference of 25% of cured cases between the two groups at 80% power and 5% level of significance.
