# Supplementary material for: An efficacy and safety report based on randomized controlled single-blinded multi-centre clinical trial of ZingiVir-H, a novel herbo-mineral formulation designed as an add-on therapy in adult patients with mild to moderate COVID-19
Source: PLoS One. 2022 Dec 6;17(12):e0276773. doi: 10.1371/journal.pone.0276773 (PMC9725144; doi:10.1371/journal.pone.0276773)
Supplement: S1 File — (DOCX) [file pone.0276773.s007.docx]

**Supplemental file**

**An efficacy and safety report based on randomized controlled single-blinded multi-centre clinical trial of ZingiVir-H, a novel herbo-mineral formulation designed as an add-on therapy in adult patients with mild to moderate coronavirus disease (COVID-19)**

Shan Sasidharan^1^*, Hareendran Nair J^1^, Srinivasakumar KP^2^, Jerin Paul^3^, Madhu Kumar R^4^, Kannan Rajendran^5^, Anita Ajit Saibannavar^6^, Sonali Nirali^7^

^1^Department of Research and Development

Pankajakasthuri Herbal Research Foundation,

Pankajakasthuri Ayurveda Medical College Campus, Killy, Kattakada, Thiruvananthapuram, Kerala, India, Pin-695572

^2^Institute of Biology and Clinical Research (IBCR),

Thiruvananthapuram, Kerala, India, Pin-695012

^3^Department of Statistics, Vimala College (Autonomous), Thrissur, Kerala, India, Pin-680009.

^4^Mysore Medical College and Research Institute, Mysuru, Karnataka, India, Pin- 570001.

^5^Saveetha Medical College & Hospital, Saveetha Nagar, Thandalam,

Chennai, India, Pin- 602105

^6^RCSM Medical College & CPR Hospital, Bhausinghaji Road, Dasara Chowk, Kolhapur District, Maharashtra, India, Pin-416002

^7^Life Point Multi Specialty Hospital, No. 145, Sr, 1, Mumbai Pune Bypass Rd, near Sayaji Hotel, Wakad, Pune, Maharashtra, India, Pin-411057

*****Corresponding author

Pankajakasthuri Herbal Research Foundation,

Pankajakasthuri Ayurveda Medical College Campus, Killy, Kattakada,

Thiruvananthapuram, Kerala, India, Pin-695572

Mail ID: [drshansasidharan@gmail.com](mailto:drshansasidharan@gmail.com)

**Document 1**

**Multi-centric study details**

Multi-centric and performed strictly in accordance with ICH-GCP; based on Clinical Trial Regulations-2019 and AYUSH guidelines. The study protocol and all other essential documents have been followed consistently in the entire four study site after the approval of respective institutional ethics committee. Please see the following details:

| **Startup Deliverables** | **Comments** |
| --- | --- |
| **PI Qualifications** | All Principal Investigators have essential residency qualifications in Modern Medicine and Experience to Conduct Clinical Trials as per ICH-GCP and Regulatory Guidelines. |
| **Documents Obtained from PI** | Signed and dated   1. Non-Disclosure Agreement 2. Financial Disclosure Form (FDA 3455) 3. Investigator Study Undertaking (FDA 1572) 4. Investigator consent on Protocol (PSP) 5. Filled in Feasibility forms 6. Investigator and study team Resume. |
| **Research support & Infrastructure at sites** | Research activities of all Identified sites was managed by Hospitals and/or through Registered Site Management Organizations in Clinical Research |
| **Research Team qualifications** | As per AYUSH Guidelines in COVID trials. |
| **Study Deliverables** | Comments |
| **Site qualification Assessments** | Online evaluation (due to COVID-19 lock down) was made based on the project feasibility submitted by the Investigator and team. Feasible sites with project execution capabilities were selected. |
| **Ethics Committee** | Documents submitted electronically to all ethics committee through Investigators. Expedite review request was made as per ICMR guidelines to Ethics Committee on COVID research studies evaluation. A few Ethics Committee gave expedite approval and four such sites were selected for the study conduct. |
| **Site Training and Initiation of the Project** | Once after Ethics Committee approval, the sites and investigation team were provided with online training about the Protocol **(details attached as annexure 1),** Study procedures including consenting research subjects and documentation, Study drug dispensing procedure, Laboratory assessments, Blood sampling procedures, Handing AE/SAE’s in the study, Data capturing requirements etcetera. |
| **Study execution and Safety measures** | The study participants are insured in the study to compensate them on any trial related injury. A study grant per patient was allocated to the site for the HR efforts. The study was conducted by a trained research team as per the delegation of responsibilities by the Chief Investigator. |
| **Study Data Collection** | Study specific Case Report forms (CRF-ATTACHED) are designed for data capture. The data points were captured in the CRF from the corresponding Patient source data by the research staff at site. CRF data were 100% audited by the Investigator and site management representative and retrieved the filled in CRF pages for data analysis. |

The study execution and data extraction, reporting, recording and transcription in CRF was performed by trained research team in all the hospitals. The study Investigator and the research coordination team were appropriately trained during site initiation and from time to time by the Clinical Trial Coordinator in the study. Further the Trial coordinator monitored and coordinated all the activities in the study to resolve the issue. These efforts made by thus leads to the successful completion of our multicenter randomized clinical without any complexity.

| **Site Number** | **Site Name and Address** | **Site Research Management Team Involved** |
| --- | --- | --- |
| Z001 | DR. Madhu Kumar, KR Hospital, Mysore Medical College and Research Institute, Mysuru | Zetalix Solutions Clinical Research, Mysuru |
| Z004 | Dr. Anita Ajit Saibannavar, RCSM Medical College & CPR Hospital, Bhausinghaji Road, Dasara Chowk, Kolhapur District, Maharashtra State, INDIA- 416002, India | CROM Clinical Research & Medical Tourism Pvt Ltd, Kolhapur |
| Z006 | DR. Kannan Rajendran, Professor in Medicine, Saveetha Medical College & Hospital, Saveetha Nagar, Thandalam, Chennai-105 | Maruti Clinical Research Services, Chennai |
| Z008 | Dr. Sonali Nirali, Consultant, Life Point Multi-Specialty Hospital, No. 145, Sr, 1, Mumbai Pune Bypass Rd, near Sayaji Hotel, Wakad, Pune, Maharashtra 411057 | Life Point Clinical Research LLP, Pune |

**Data collection and coordination**

The study is coordinated and the site-specific training was managed by Institute of Biology and Clinical Research (IBCR) based in Thiruvananthapuram. [www.ibcrglobal.org](http://www.ibcrglobal.org)

| **Study Deliverables** | **Comments** |
| --- | --- |
| **Site Training and Initiation of the Project** | Once after Ethics Committee approval the sites and investigation team are provided with telephonic training on Protocol, Study procedures including consenting research subjects and documentation, Study drug dispensing procedure, Laboratory assessments, Blood sampling procedures, Handing AE/SAE’s in the study, Data capturing requirements etcetera. |
| **Study Data Collection** | Study specific Case Report forms (CRF) are designed for data capture. The data points were captured in the CRF from the corresponding Patient source data by the research staff at site. CRF data were 100% audited by the Investigator and site management representative and retrieved the filled in CRF pages for data analysis. |
| **Record of Site Training** | Site Training Log signed by Principal / CO Investigator (Training log attached as annexure 1) |

**Data Monitoring Committee (DMC)**

Data Monitoring Committee (DMC) for Protocol number PHRF 010-2020 with Title Randomized controlled Single blinded prospective multi centre clinical trial to investigate the safety and efficacy of ZingiVir-H as an adjuvant therapy in hospitalized adults diagnosed with coronavirus disease 2019 (COVID-19) formed on 11 April 2020. The Charter defined the primary responsibilities of the DMC, its relationship with other trial components, its membership, and the purpose and timing of its meetings. The Charter also provide the procedures for ensuring confidentiality and proper communication, the statistical monitoring guidelines implemented by the DMC, and an outline of the content of the Open and Closed Reports that provided to the DMC.

The DMC appointed will be responsible for

- Safeguarding the interests of trial participants, assessing the safety and efficacy of the interventions during the trial.
- Monitoring the overall conduct of the clinical trial.
- To provide recommendations about stopping or continuing the trial.
- To contribute to enhancing the integrity of the trial,
- DMC may also formulate recommendations relating to the selection/ recruitment/ retention of participants, their management, improving adherence to protocol-specified regimens and retention of participants, and the procedures for data management and quality control.

The DMC are advisory to the clinical trial leadership Steering Committee (SC) group and usually including sponsor representatives. The SC will be responsible for promptly reviewing the DMC recommendations, to decide whether to continue or terminate the trial, and to determine whether amendments to the protocol or changes in study conduct are required. The Clinical Research experts as consultants to the sponsor for the study are the member of Steering Committee.

**Document 2**

**Sample Size computation for comparison of two proportion**

The equation for the calculation of sample size for the comparison of two proportion is given by

$$n=\frac{2 p q {(Z_{1-\frac{\alpha}{2}}+Z_{1-\beta})}^{2}}{{(p_{1}-p_{2})}^{2}}$$

The terms involved in this equation are explained below

In this study we have two groups viz, placebo control and case group.

| Placebo-Control | Placebo-Control | Cases | Cases |
| --- | --- | --- | --- |
| % of Cured (PCR Negative) | % of Non-cured (PCR Positive) | % of Cured (PCR Negative) | % of Non-cured (PCR Positive) |
| p_1_ | q_1_ | p_2_ | q_2_ |

$p=\frac{p_{1}+p_{2}}{2}$ and $q=\frac{q_{1}+q_{2}}{2}$

$Z_{1-\frac{\alpha}{2}}$ is the Z value at ‘α’ level of significance

| **Level of significance** | $Z_{1-\frac{\alpha}{2}}$ **Value** |
| --- | --- |
| 5% | 1.96 |
| 1% | 2.58 |

$Z_{1-\beta}$ is the Z value at (1-β)% power

| **Power** | $Z_{1-\beta}$ **Value** |
| --- | --- |
| 80% | 0.84 |
| 90% | 1.28 |

$p_{1}-p_{2}$ sometimes notated by *d* known as clinically significance difference (Minimum difference (between the groups) which the investigator considers as clinically relevant and it is decided by the investigator)

For this study specifically I have taken the following values

$$p_{1}=0.6, p_{2}=0.85, q_{1}=0.4, q_{2}=0.15, p=0.725, q=0.275, and p_{1}-p_{2}=-0.25$$

$Z_{1-\frac{\alpha}{2}}=1.96$ at ‘5%’ level of significance

$Z_{1-\beta}=0.84$ at 80% power

Now

$$n=\frac{2*0.725*0.275*{(1.96+0.84)}^{2}}{{(-0.25)}^{2}}=48.82641$$

A 10% dropout is expecting, which will be equal to $= 0.1*48.82641 = 4.882641$

Therefor the total sample size $= 48.82641+4.882641=53.70905\approx54$

Approximately 54 samples should be required in each group to detect a clinically significance difference of 25% of cured cases between the two groups at 80% power and 5% level of significance.
