# Supplementary material for: An efficacy and safety report based on randomized controlled single-blinded multi-centre clinical trial of ZingiVir-H, a novel herbo-mineral formulation designed as an add-on therapy in adult patients with mild to moderate COVID-19
Source: PLoS One. 2022 Dec 6;17(12):e0276773. doi: 10.1371/journal.pone.0276773 (PMC9725144; doi:10.1371/journal.pone.0276773)
Supplement: S2 File — (DOCX) [file pone.0276773.s008.docx]

**RANDOMIZED CONTROLLED SINGLE BLINDED PROSPECTIVE MULTI CENTRE CLINICAL TRIAL TO INVESTIGATE THE SAFETY AND EFFICACY OF ZINGIVIR-H AS AN ADJUVANT THERAPY IN HOSPITALIZED ADULTS DIAGNOSED WITH CORONAVIRUS DISEASE 2019 (COVID-19).**

**Protocol Number: PHRF 010-2020**

**Version 1:1**

**(ADDENDUM)**

**Final Protocol: 11 April 2020**

**Addendum to final Protocol dated: 19 May 2020 (2 Pages)**

**Drug License No: 50/25D/96**

| **Study Sponsor** | Pankajakasthuri Herbal Research Foundation  Poovachal, Thiruvananthapuram-695575, Kerala. |
| --- | --- |

| **DR. J. HAREENDRAN NAIR**    STUDY DIRECTOR | **Signature………………………………** |
| --- | --- |
| **DR. SREEKUMAR.G.S**  MEDICAL MONITOR | **DR. PREM KIRAN**  MEDICAL MONITOR |
| **DR. K.P. SRINIVASAKUMAR**  CLINICAL RESEARCH COORDINATOR | **DR. SHAN SASHIDHARAN**  PRODUCT DEVELOPMENT I/C |

**ADDENDUM TO CSP PHRF 010-2020**

As per the recent notification issued by Ministry of Health & Family Welfare, Directorate General of Health Services, EMR Division regarding the Guidance document on appropriate management of suspect/confirmed cases of COVID-19 which waives off that there will be no need for testing RT-PCR in patients prior to discharge for "mild/very mild/pre-symptomatic" cases of COVID-19. Such patients can be discharged after 10 days of symptom onset and absence of fever for the last three days. "At the time of discharge, the patient will be advised to follow the home isolation for further seven days.

Ref: <https://www.mohfw.gov.in/pdf/FinalGuidanceonMangaementofCovidcasesversion2.pdf>

Considering this guideline, the following procedure is included in the parent protocol PHRF-010-2020 version 1.1 dated 11 April 2020. An RT-PCR test schedule shall be included in the protocol for the study participants to understand the primary endpoint of our study. Accordingly RTPCR test can be performed on the 4th, 8th, 12th & 15th day of the study period start from the day of enrollment (Day-01) until the study subjects shown negative to COVID test. The RT-PCR sampling can also be performed additionally on any days as per Investigator discretion. Day-01 stands at the day the subjects started consuming the study drug OR Placebo. The test sampling shall be withdrawn as per the existing sampling for COVID diagnosis protocol.

Secondly as per the protocol Interleukin-7 test (IL-7) was considered as Inflammatory markers to access the efficacy, which is waived off as per the present addendum. As Interleukin-7 (IL-7) is not considered as significant inflammatory markers as per review of literature, the test is waived off in the protocol and no protocol deviation shall be filed on this behalf at study level. As per laboratory procedures only CBC, CRP, LFT, RFT, IL6, IgG and IgM shall be performed as per study procedures.

Also Masking criteria is considered as per Single blinded protocol whereas the trial participants and LAR if applicable ONLY are blinded from the study group information.
