# Supplementary material for: An efficacy and safety report based on randomized controlled single-blinded multi-centre clinical trial of ZingiVir-H, a novel herbo-mineral formulation designed as an add-on therapy in adult patients with mild to moderate COVID-19
Source: PLoS One. 2022 Dec 6;17(12):e0276773. doi: 10.1371/journal.pone.0276773 (PMC9725144; doi:10.1371/journal.pone.0276773)
Supplement: S3 File — (DOCX) [file pone.0276773.s009.docx]

**RANDOMIZED CONTROLLED SINGLE BLINDED PROSPECTIVE MULTI CENTRE CLINICAL TRIAL TO INVESTIGATE THE SAFETY AND EFFICACY OF ZINGIVIR-H AS AN ADJUVANT THERAPY IN HOSPITALIZED ADULTS DIAGNOSED WITH CORONAVIRUS DISEASE 2019 (COVID-19).**

**Protocol Number: PHRF 010-2020**

**Version 1:1**

**Final: 11 April 2020**

**Drug License No: 50/25D/96**

| **Study Sponsor** | Pankajakasthuri Herbal Research Foundation  Poovachal, Thiruvananthapuram-695575, Kerala. |
| --- | --- |

| **DR. J. HAREENDRAN NAIR**    STUDY DIRECTOR | **Signature………………………………** |
| --- | --- |
| **DR. SREEKUMAR.G.S**  MEDICAL MONITOR | **DR. PREM KIRAN**  MEDICAL MONITOR |
| **DR. K.P. SRINIVASAKUMAR**  CLINICAL RESEARCH COORDINATOR | **DR. SHAN SASHIDHARAN**  PRODUCT DEVELOPMENT I/C |

**Protocol Synopsis**

**Title of Study:** Randomized controlled Single blinded prospective multi centre clinical trial to investigate the safety and efficacy of ZingiVir-H as an adjuvant therapy in hospitalized adults diagnosed with coronavirus disease 2019 (COVID-19).

**Name of Sponsor/Company:** Pankajakasthuri Herbal Research Foundation, Thiruvananthapuram, Kerala, India

**Name of Investigational Product:** ZingiVir-H

**Name of Active Ingredient:** Herbal ingredient extracted from Lavanga (*Eugenia caryophyllus*), Ardraka (*Zingiber officianale*), Mustha (*Cyperus rotundus*), Parpataka (*Hedyotis corymbosa*), Ajamoda (*Trachyspermum ammi*), Hingula (Cinnabar, HgS), Haratala (Arsenic trisulphide)

**Objectives**

**Primary:**

- The primary objective of this study is to evaluate the efficacy of ZingiVir-H regimens and standard of care compared to placebo and standard of care (SOC), with respect to clinical status assessed based on patient diary data and based on Ordinal scale on the ratio of improvement. [Time Frame: From the time of randomization until the time of alleviation of all symptoms, assessed up to the test results shows negative]
- To determine the safety of ZingiVir-H in this test population.

**Secondary:**

- Time to a normal body temperature [ Time Frame: From the time of randomization until the time of normal body temperature, assessed up to 15 days]
- Time to the alleviation of nasal/throat/chest symptoms [ Time Frame: From the time of randomization until the time of absence of nasal/throat/chest symptoms, assessed up to 15 days
- Proportion of participants in each group with normalization of fever [ Time Frame: day 7 and day 15 ]
- Proportion of participants in each group with oxygen saturation > 94% on room air for >24h [ Time Frame: day 7 and day 15 ]
- Time to fever normalization (if febrile at baseline) [ Time Frame: within 15 days ]
- Time to first negative SARS-CoV-2 PCR in NP swap (if pos. at baseline) [ Time Frame: within 15 days ]
- Time to first negative SARS-CoV-2 PCR in lower respiratory tract specimens (sputum, bronchoalveolar lavage, tracheal aspirate) (if positive at baseline) [ Time Frame: within 15 days ]
- Duration of oxygen therapy [ Time Frame: within 30 days ]
- Proportion of participants in each group with need for mechanical ventilation [ Time Frame: within 30 days ]
- Duration of hospitalization [ Time Frame: within 28 days ]
- All cause mortality [ Time Frame: day 30 ]

**Study Center(s):** Study in Indian population and recruitment will happen up to 5 research sites based on competitive patient recruitment.

**Phase of Development:** Proof of Concept/ Intervention Trial on traditional medical product.

**Study Period:**

Estimated time of first subject enrollment: 1 Q 2020

Estimated time of last subject enrollment: 1Q 2020

Number of Subjects (planned): Approximately 112 randomized subjects with assumed drop-outs of 10%; 100 evaluable subjects.

**Diagnosis and Main Criteria for Inclusion of Study Subjects:**

This study targets infectious subjects normally associated with treatment in hospital setting. The following main criteria for inclusion must be met for study entry:

- Patients of both sexes aged from 18 years to 60 years old.
- Willing and able to provide written informed consent prior to performing study procedures by the subject or legal guardian willing and able to provide written informed consent prior to performing study procedures
- Patients with Severe Acute Respiratory Syndrome Coronavirus (SARS-CoV)-2 infection confirmed by RT Polymerase chain reaction (RT-PCR) test between 1 and 120 Hours before randomization
- Currently hospitalized and requiring medical care for COVID-19
- Peripheral capillary oxygen saturation (SpO2) > 94% on room air at screening
- Radiographic evidence of pulmonary infiltrates

**Investigational Product, manufacturing, Dosage and Mode of Administration:**

The study drug ZingiVir-H is a poly herbo-mineral formulation, which is a blend of antiviral phyto-molecules and processed purified and detoxified form of natural metallic compounds. It is a unique combination of Lavanga (Eugenia caryophyllus), Ardraka (Zingiber officianale), Mustha (Cyperus rotundus), Parpataka (Hedyotis corymbosa), Ajamoda (Trachyspermum ammi), Hingula (Cinnabar, HgS), Haratala (Arsenic trisulphide) and Pharmaceutical grade starch. Even though the study drug contains minerals such as HgS and As2S3 their dose is designed in such a way that there is no cumulative dose sufficient for making any type of toxicity in the continuous usage for maximum of 15 days. The drugs in the combination have showed marked antiviral, antipyretic and immunomodulatory effects.

**Therapeutic dose**:

ONE tablet (500 mg) each consumed once in 3 hours ±1 hour between 6 AM and 9 PM in a given day (6AM, 9AM, 12Noon, 3PM, 6PM, 9PM) for a minimum duration of 10 days to Maximum 15 days as per the clinical conditions and disease outcome.

**Masking:**

Quadruple (Participant, Care Provider, Investigator, Outcomes Assessor)

**Duration of Treatment**: Fifteen (15) days of study drug regimen and a follow-up on 30^th^ day

**Reference Therapy, Dosage and Mode of Administration**: Placebo and Standard of Care (SOC)

Placebo for this study is supplied as identically labeled, tablets. Placebo will also be required to be maintained in controlled environment as IP. Placebo must be consumed according to the same procedures and guidelines as ZingiVir-H.

**Primary Efficacy Endpoint:**

The Odds of Ratio for Improvement on a 7-point Ordinal Scale on Day 15 [Time Frame: Day 15 from the day of study inclusion]. The odds ratio represents the odds of improvement in the ordinal scale between the treatment groups. The ordinal scale is an assessment of the clinical status at a given day. Each day, the worst score from the previous day will be recorded.

The scale is as follows: 1. Death 2. Hospitalized, on invasive mechanical ventilation or Extracorporeal Membrane Oxygenation (ECMO) 3. Hospitalized, on non-invasive ventilation or high flow oxygen devices 4. Hospitalized, requiring low flow supplemental oxygen 5. Hospitalized, not requiring supplemental oxygen - requiring ongoing medical care (Coronavirus (COVID-19) related or otherwise) 6. Hospitalized, not requiring supplemental oxygen - no longer required ongoing medical care (other than per protocol ZingiVir-H administration) 7. Discharge from hospital.

**Secondary Efficacy Endpoint:**

- Proportion of Participants experiencing Treatment-Emergent Adverse Events [Time Frame: First dose date up to 15 days]
- Changes in Haematological and Biochemical parameter like CBC,ESR, CRP, Troponin, Liver function test, Renal function test, [Time Frame: First dose date up to 15 days]
- Changes in inflammatory markers - IL2, IL6, Interferon α, IgG, IgM
- Significant changes in the Chest X-Ray before and after treatment.

**Primary Safety Endpoints:**

- Serious Adverse Events
- Adverse Events
- Respiratory failure (Hypercapnic)

**Statistical Methods:**

The total sample size is 112 randomized subjects. This sample size is allocated to treatment arms in a 1 to 1 ratio. This sample size provides 50% power if the following assumptions are made based on earlier reviews. SAS 9.4 statistical analysis software will be used for statistical analyses. T-test will be used for comparisons between groups for continuous variables. The comparison between groups for categorical variables will be performed using Fisher's exact probability method. Means and standard deviations will be used to describe centralized discrete trends. The t-test, rank-sum test, and Fisher's exact probability method will be used for comparisons between groups. P 0.05 indicates that the difference was statistically significant.

**RANDOMIZED CONTROLLED SINGLE BLINDED PROSPECTIVE MULTI CENTRE CLINICAL TRIAL TO INVESTIGATE THE SAFETY AND EFFICACY OF ZINGIVIR-H AS AN ADJUVANT THERAPY IN HOSPITALIZED ADULTS DIAGNOSED WITH CORONAVIRUS DISEASE 2019 (COVID-19).**

**Version 1:1 Dated 11 April 2020**

**PROTOCOL APPROVAL**

The signature of the Principal Investigator constitutes an agreement that this study will be conducted according to all stipulations, clinically and administratively, as stated in the protocol, including all statements as to confidentiality. It is agreed that the conduct and results of this study will be kept confidential and that the case report forms and other pertinent data will become the property of Pankajakasthuri Herbal Research Foundation, Thiruvananthapuram.

It is agreed that the protocol contains all necessary information required to conduct the study as outlined in the protocol, and that the study will not be initiated without the approval of an appropriate Institutional Review Board or Ethics Review Committee.

It is agreed that all participants in this study will provide written informed consent in accordance with ICH Guidelines for Good Clinical Practice and the requirements specified in the Code of Federal Regulations (21 CFR Parts 50, 56, 312) and/or the Declaration of Helsinki. All participants will also be informed that their medical records will be kept confidential except for review by authorized representatives of Pankajakasthuri Herbal Research Foundation and its associates, the DCGI, ICMR or other regulatory agencies.

PRINCIPAL INVESTIGATOR SIGNATURE DATE

By signing the above I agree to perform the study in accordance with the protocol, ICH Good Clinical Practice (GCP) guidelines, and all applicable regulations.

**BACKGROUND:**

The epidemic of SARS-CoV-2, causing the disease COVID-19 has expanded from Wuhan throughout China and is being exported to a growing number of countries including India, some of which have seen onward transmission. As per WHO total number of confirmed cases are 1,714,782 and 103,803 number of deaths were reported, and in India 5,734 corona virus cases and 166 deaths has been reported till April 11, 2020. In India COVID-19 causes serious threat with over 6634 confirmed cases with 242 deaths in the same timeline. The disease has shown to put an enormous burden on the society and is a challenge to the physician owing to its infectivity, complications, morbidity and absence of proper medication. As medicine is an ever-changing science new research and clinical experience broaden our knowledge and bring changes in drug therapy. COVID-19 is now spreading throughout the world because of its high infectivity and communicability. Also there is a lack in the proper management of the disease. So any contribution in the knowledge or treatment of COVID-19 from any source could be of great value to the medical profession.

**INTRODUCTION**

Viruses are responsible for a number of human pathogeneses including cancer. Several hard-to-cure diseases and complex syndromes including Alzheimer's disease, type 1 diabetes, and hepatocellular carcinoma have been associated with viral infections. Moreover, due to increased global travel and rapid urbanization, epidemic outbreaks caused by emerging and re-emerging viruses represent a critical threat to public health, particularly when preventive vaccines and antiviral therapies are unavailable. Examples include the recent emergence of dengue virus, influenza virus, measles virus, severe acute respiratory syndrome (SARS) virus, and West Nile virus outbreaks. To date, however, many viruses remain without effective immunization and only few antiviral drugs are licensed for clinical practice. The situation is further exacerbated by the potential development of drug-resistant mutants, especially when using viral enzyme-specific inhibitors, which significantly hampers drug efficacy. Hence, there is an urgent need to discover novel antivirals that are highly efficacious and cost-effective for the management and control of viral infections when vaccines and standard therapies are lacking. Due to the rapid emergence of deadly virus strains worldwide, the development of effective therapies for human viral diseases has become an emergency necessity. This is mostly dependent upon the identification of novel therapeutic agents with low toxicity. It is reported that plants have been used in medicines for thousands of years; in particular traditional medicines. Herbal medicines and purified natural products provide a rich resource for novel antiviral drug development. Identification of the antiviral mechanisms from these natural agents has shed light on where they interact with the viral life cycle, such as viral entry, replication, assembly, and release, as well as on the targeting of virus–host-specific interactions. These traditional medicines employing natural products have shown to contain antiviral compounds in vitro. The treasure troves of Ayurvedic & Siddha references and experiences passed over many decades channeled the development of a poly herbomineral formulation, which is a blend of antiviral phytomolecules and processed, purified, and detoxified form of natural metallic compounds. Under the stringent quality control process and GMP protocols, thus formulated products show significant antiviral property majorly by three mechanisms by

1. Inhibiting viral attachment, internalization, stimulating IFN-α secretion

2. Inhibiting the replication of the virus by producing an enhanced level of glutathione.

3. Inhibiting all proteases in the whole process of viral replication, and inactivating these proteases thus destructing the virus.

It is scientifically proven for its antipyretic, anti-inflammatory, and anti-bronchitis activities. The combined effect will reduce the viral infection and associated symptoms without any side effects. As the course duration is very small, just below a couple of weeks, the toxicity issues are out of curiosity. The formulation can play a vital role in therapeutics as a main antiviral agent or as catalysing and supporting therapeutic agents in antiviral therapy.

The study drug is a poly herbo-mineral formulation, which is a blend of antiviral phyto-molecules and processed purified and detoxified form of natural metallic compounds. It is a unique combination of *Lavanga (Eugenia caryophyllus), Ardraka (Zingiber officianale), Mustha (Cyperus rotundus), Parpataka (Hedyotis corymbosa), Ajamoda (Trachyspermum ammi), Hingula (Cinnabar, HgS), Haratala (Arsenic trisulphide)* and Pharmaceutical grade starch. Even though the study drug contains minerals such as HgS and As_2_S_3_ their dose is designed in such a way that there is no cumulative dose sufficient for making any type of toxicity in the continuous usage for maximum of 15 days. The drugs in the combination have showed marked antiviral, antipyretic and immune-modulatory effects. Hence it will be a great benefit to the humanity, if this drug is brought to the main stream of management.

**INVESTIGATIONAL PLAN**

**Overall Study Design**

This is a Randomized controlled, single blinded multi-center, comparative study of ZingiVir-H as an adjuvant therapy in hospitalized adults diagnosed with coronavirus disease 2019 (COVID-19). At inclusion, subjects who meet all inclusion criteria and no exclusion criteria will be assigned, in either therapeutic arm (ZingiVir-H), to receive ZingiVir-H in a dose of 500 mg tablet each consumed once in 3 hours ±1 hour between 6 AM and 9 PM in a given day (6AM, 9AM, 12Noon, 3PM, 6PM, 9PM) for a minimum duration of 10 days to Maximum 15 days OR will be on a Placebo arm to receive placebo (without active ingredients) each consumed once in 3 hours ±1 hour between 6 AM and 9 PM in a given day (6AM, 9AM, 12Noon, 3PM, 6PM, 9PM) for a minimum duration of 10 days to Maximum 15 days. The allocation of study arm is strictly as per the discretion of the Investigator. Whereas both the study groups shall be given standard of care of treatment as per the research site policies or COVID-19 therapeutic management policies. These doses will be administered orally. Blood samples for assessing CBC; safety laboratory tests and immunomodulatory markers will be obtained between baseline assessments (Day-01), during the day-06 of inclusion, at the end of the treatment day-15 and at the end of the study day-30. Safety-related assessments will include reports of adverse events and clinical laboratory test results.

**Study Objectives**

**Primary:**

- The primary objective of this study is to evaluate the efficacy of ZingiVir-H regimens and standard of care compared to placebo and standard of care (SOC), with respect to clinical status assessed based on patient diary data and based on Ordinal scale on the ration of Improvement. [Time Frame: From the time of randomization until the time of alleviation of all symptoms, assessed up to the test results shows negative]
- To determine the safety of ZingiVir-H in this test population.

**Secondary:**

- Time to a normal body temperature [ Time Frame: From the time of randomization until the time of normal body temperature, assessed up to 15 days]
- Time to the alleviation of nasal/throat/chest symptoms [ Time Frame: From the time of randomization until the time of absence of nasal/throat/chest symptoms, assessed up to 15 days
- Proportion of participants in each group with normalization of fever [ Time Frame: day 7 and day 15 ]
- Proportion of participants in each group with oxygen saturation > 94% on room air for >24h [ Time Frame: day 7 and day 15 ]
- Time to fever normalization (if febrile at baseline) [ Time Frame: within 15 days ]
- Time to first negative SARS-CoV-2 PCR in NP swap (if pos. at baseline) [ Time Frame: within 15 days ]
- Time to first negative SARS-CoV-2 PCR in lower respiratory tract specimens (sputum, bronchoalveolar lavage, tracheal aspirate) (if positive at baseline) [ Time Frame: within 15 days ]
- Duration of oxygen therapy [ Time Frame: within 30 days ]
- Proportion of participants in each group with need for mechanical ventilation [ Time Frame: within 30 days ]
- Duration of hospitalization [ Time Frame: within 28 days ]
- All cause mortality [ Time Frame: day 30 ]

**Study Population**

Approximately 112 subjects will be randomized and considering 10% drop-out, the final evaluable subjects shall be 100.

**Subject Eligibility Criteria**

**Inclusion Criteria**

This study targets infectious subjects normally associated with treatment in hospital setting. The following main criteria for inclusion must be met for study entry:

1. Patients of both sexes aged from 18 years to 60 years old.
2. Willing and able to provide written informed consent prior to performing study procedures by the subject or legal guardian willing and able to provide written informed consent prior to performing study procedures
3. Patients with Severe Acute Respiratory Syndrome Coronavirus (SARS-CoV)-2 infection confirmed by RT Polymerase chain reaction (RT-PCR) test between 1 and 120 Hours before randomization
4. Currently hospitalized and requiring medical care for COVID-19
5. Peripheral capillary oxygen saturation (SpO2) > 94% on room air at screening
6. Radiographic evidence of pulmonary infiltrates

**Exclusion Criteria**

Candidates for the study will be excluded if ANY of the following criteria are present:

1. Subject or Authorized Representative is unable to provide informed consent
2. Subject is pregnant or breastfeeding ladies
3. Subject is of childbearing potential and has a positive pregnancy test since admission to the hospital
4. Subject is < 18 years of age
5. Subject has a known allergy to herbal compounds or ZingiVir-H or any components of the drug product
6. Subject has had previous treatment with ZingiVir-H
7. Body weight ≥ 175 kg
8. Intra-thoracic or intra-abdominal surgery within the 12 hours prior to consent, or ongoing impairment of hemostasis as a result of one of these procedures
9. A history of head trauma, spinal trauma, or other acute trauma with an increased risk of bleeding.
10. Cerebral Vascular Accident (CVA) or Intracerebral Arteriovenous Malformation (AVM), cerebral aneurysm, or mass lesions of the central nervous system or melena, hematemesis.
11. Inability to take oral medication
12. Prolonged QTc-interval in baseline ECG (>500 ms)
13. History of solid organ, allogeneic bone marrow, or stem cell transplantation.
14. Severe renal failure characterized by chronic or acute need of hemodialysis, hemofiltration or peritoneal dialysis
15. Need of anticoagulants, antiplatelet agents, antithrombotics and thrombolytics during the treatment period.
16. Participation in another research study involving an investigational agent within 30 days prior to consent

**Criteria for Discontinuation from Study Drug or Study Participation**

**Withdrawal from receiving study drug**

A subject may remain in the study but discontinue the administration of study drug at any time during treatment. Reasons for this may include:

- Intolerable adverse event(s)
- Subject’s request
- Investigator’s judgment (e.g. related SAE)
- Administration of study drug is contraindicated if the co-administration of standard of care medication which is required for subject treatment.
- Need for mechanical intubation due to respiratory failure (Hypecapnic)

In any above case of withdrawal or lost to follow-up on the patients status shall be document as appropriate in the patient source document and needs to be reported as appropriate to sponsors.

**Investigational Product, manufacturing, Dosage and Mode of Administration: ZingiVir-H**

The study drug ZingiVir-H is a poly herbo-mineral formulation, which is a blend of antiviral phyto-molecules and processed purified and detoxified form of natural metallic compounds. It is a unique combination of Lavanga (Eugenia caryophyllus), Ardraka (Zingiber officianale), Mustha (Cyperus rotundus), Parpataka (Hedyotis corymbosa), Ajamoda (Trachyspermum ammi), Hingula (Cinnabar, HgS), Haratala (Arsenic trisulphide) and Pharmaceutical grade starch. Even though the study drug contains minerals such as HgS and As2S3 their dose is designed in such a way that there is no cumulative dose sufficient for making any type of toxicity in the continuous usage for maximum of 15 days. The drugs in the combination have showed marked antiviral, antipyretic and immunomodulatory effects.

**Therapeutic dose**:

ONE tablet (500 mg each consumed once in 3 hours ±1 hour between 6 AM and 9 PM in a given day (6AM, 9AM, 12Noon, 3PM, 6PM, 9PM) for a minimum duration of 10 days to Maximum 15 days continuously as per the clinical conditions and disease outcome. Compliance on study drug consumption is important to evaluate the study endpoints.

**Masking:**

Quadruple (Participant, Care Provider, Investigator, Outcomes Assessor)

**Duration of Treatment**: Minimum of Ten days to Maximum of Fifteen (15) days of study drug regimen and a follow-up on 30^th^ day from the day of baseline.

**Reference Therapy, Dosage and Mode of Administration**: Placebo and Standard of Care (SOC)

Placebo for this study is supplied as identically labeled, tablets. Placebo will also be required to be maintained in controlled environment as IP. Placebo must be consumed according to the same procedures and guidelines as ZingiVir-H.

**Blinding**

Study drug will be assigned and administered in a Single blind fashion.

In the event of a medical emergency where it is imperative to know which treatment arm the subject was randomized to in order to make future treatment decisions, the Investigator may break the blind for an individual subject after getting approval from study monitor or sponsor. If any adverse events causing the need for subject unblinding, will be handled as a serious adverse event in accordance with the procedures outlined in this protocol. Any broken blind will need to be clearly justified, explained by a comment within the Case Report Form (CRF), and captured on the Serious Adverse Event Form.

**Laboratory Samples**

Hospital laboratory can be used to access the following test. If the hospital laboratory has limitation an outside laboratory with NABL accreditations shall be arranged for sample analysis.

- Complete Blood Count with differential (Hemoglobin, Hematocrit, and WBC with differential count) & ESR – **Day-01, 06, EOT (Day15) & 30 of study period**
- Serum Chemistry- Creatinine, Urea, CRP, ALT, AST, Alkaline Phosphatase, Albumin, total bilirubin- **Day-01, Day 06 Only CRP, End of treatment- Day 10 OR Day 15, & End of study period Day 30**.
- Pregnancy test; Serum or Urine human Chorionic Gonadotropin (hCG) - Pregnancy test only required for woman of childbearing potential on the day of randomization (**Day-01**).
- Immunomodulatory markers, IL6, IL7, Interferon α, IgG, IgM- **Day 01, Day 06, End of treatment- Day 10 OR Day 15, & End of study period Day 30**.

**Visit Schedule and Assessments**

**Screening / Randomization Visit – Day 01**

Subjects must meet the inclusion criteria and have none of the exclusion criteria in order to be eligible for randomization in this study. Subjects who meet all eligibility criteria, and have signed informed consent will be discussed randomize and move on to the Baseline study visit. Once the subject is randomized the sponsor representative or the study Investigator will provide the study staff with a unique randomization number for the subject.

Following assessments must be made at this Baseline visit for each study subject:

- RT-PCR test assuring the subjects test positive for COVID-19.
- Demographics
- Medical history (all known information regarding the subject’s health history/relevant surgeries/interventions prior to signing the informed consent).
- Vital signs: Body temperature, Pulse – beats per minute (BPM), Blood Pressure (BP) mmHg, Respirations – breaths per
- Concomitant medications (including medications, fluids and blood products administered within the 7 days prior to randomization).
- Documentation of any other infection if available (site of infection and, if available: positive culture results, prior antibiotics, sensitivity of cultured organism to antibiotics, nonmedical treatment such as surgery or drainage).
- Physical Examination including the following: general appearance, Head, Eye, Ear Nose Throat (HEENT), neck, respiratory, cardiovascular, chest, abdomen, lymphatic, musculoskeletal and extremities, skin and neurologic examination. Height and weight (baseline dry weight).
- Blood will be obtained for following laboratory tests:
  - Complete Blood Count with differential (Hemoglobin, Hematocrit, and WBC with differential count) & ESR
  - Serum Chemistry- Creatinine, Urea, CRP, ALT, AST, Alkaline Phosphatase, Albumin, total bilirubin
  - Pregnancy test; Serum or Urine human Chorionic Gonadotropin (hCG) - Pregnancy test only required for woman of childbearing potential on the day of randomization
  - Immunomodulatory markers, IL6, IL7, Interferon α, IgG, IgM

**Dosing Visits - Day-1 to EOT (Day15)**

The first dose of study drug must be swallowed within 4 hours from the time study inclusion. The study drug shall be continuing daily each consumed once in 3 hours ±1 hour between 6 AM and 9 PM in a given day (6AM, 9AM, 12Noon, 3PM, 6PM, 9PM) for a minimum duration of 10 days to Maximum 15 days whereas the daily dose shall be administered orally once in 4 ±2 hours interval ie 6 tablets per day.

Following the administration of the first dose record the following:

- Changes in concomitant medications
- Adverse Events/Serious Adverse Events
- Major respiratory failure Events
- Interventions or Surgeries
- Vital signs

**Routine assessment visit and Study drug compliance** – Day 2 to EOT (Day 15)

Following assessments must be made on daily basis for each study subject:

- Vital signs: Pulse – Body temperature, beats per minute (BPM), Blood Pressure (BP) mmHg, Respirations – breaths per minute
- Concomitant medications (including medications, fluids and blood products administered within the 7 days prior to randomization).
- Documentation of any adverse events
- Blood will be obtained for following laboratory tests – only on **Day 06**
  - Complete Blood Count with differential (Hemoglobin, Hematocrit, and WBC with differential count)
  - Serum Chemistry ESR, CRP
  - Immunomodulatory markers, IL6, IL7, Interferon α, IgG, IgM

**End of treatment visit (EOT) - Day 15**

Following assessments must be made at this EOT visit for each study subject:

- Demographics
- Vital signs: Pulse – Body temperature, beats per minute (BPM), Blood Pressure (BP) mmHg, Respirations – breaths per
- Concomitant medications and Prescribed medications
- Physical Examination including the following: general appearance, Head, Eye, Ear Nose Throat (HEENT), neck, respiratory, cardiovascular, chest, abdomen, lymphatic, musculoskeletal and extremities, skin and neurologic examination.
- Blood will be obtained for following laboratory tests:
  - Complete Blood Count with differential (Hemoglobin, Hematocrit, and WBC with differential count) & ESR
  - Serum Chemistry- Creatinine, Urea, CRP, ALT, AST, Alkaline Phosphatase, Albumin, total bilirubin
  - Immunomodulatory markers, IL6, IL7, Interferon α, IgG, IgM

**End of Study visit (Day 30 ± 2 days) –Follow up**

- Changes in concomitant medications
- Adverse Events/Serious Adverse Events
- Vital signs
- Blood will be obtained for following laboratory tests once in 24 ±2 hours:
  - Complete Blood Count with differential (Hemoglobin, Hematocrit, and WBC with differential count) & ESR
  - Serum Chemistry- Creatinine, Urea, CRP, ALT, AST, Alkaline Phosphatase, Albumin, total bilirubin
  - Immunomodulatory markers, IL6, IL7, Interferon α, IgG, IgM

**Ordinal Scale assessment: Day 01 to EOT & EOS**

Ordinal Scale on the Odds of Ratio of Improvement on a 7-point scale between the treatment groups. The ordinal scale is an assessment of the clinical status will be assessed and documented on daily basis from Day-01 to EOT and on EOS. Each day, the worst score from the previous day will be recorded in the CRF.

The scale is as follows: 1. Death 2. Hospitalized, on invasive mechanical ventilation or Extracorporeal Membrane Oxygenation (ECMO) 3. Hospitalized, on non-invasive ventilation or high flow oxygen devices 4. Hospitalized, requiring low flow supplemental oxygen 5. Hospitalized, not requiring supplemental oxygen - requiring ongoing medical care (Coronavirus (COVID-19) related or otherwise) 6. Hospitalized, not requiring supplemental oxygen - no longer required ongoing medical care (other than per protocol ZingiVir-H administration) 7. Discharge from hospital.

**Safety Assessments and Reporting**

**Adverse Events**

An Adverse Event (AE) is defined as any untoward medical occurrence in a subject administered a pharmaceutical product, which does not necessarily have a causal relationship with this treatment. An AE can therefore, be any unfavorable and unintended sign, symptom, or disease temporally associated with the use of a medicinal (investigational) product, whether or not considered related to the medicinal (investigational) product. Examples of adverse events include:

- Increase in severity of a baseline event
- Clinically significant worsening of lab values post baseline
- A new event with an onset date post Baseline

As far as possible, each AE will also be described by:

- The duration (start and end dates)
- The severity grade (Grade 1/mild, Grade 2/moderate, Grade 3/severe, Grade 4/Potentially life threatening as defined by the DAIDS Toxicity Table)
- The relationship to the study drug (none, unlikely, related)
- Whether the AE involved bleeding of any kind
- The action(s) taken
- The outcome

**Serious Adverse Events**

Information about all serious adverse events (SAEs) will be collected and recorded on the SAE Report form within 24 hours of learning of its occurrence. A paper SAE form should be completed by the site and faxed or emailed to the sponsor within 24 hours using the safety reporting contact information provided. The site is to follow their local regulations related to the reporting of SAEs to their local IRB/IEC. An SAE is any undesirable sign, symptom, or medical condition which:

• Is fatal or life-threatening (i.e. an event with an outcome of ‘Death’), or

• Requires or prolongs inpatient hospitalization, or

• Results in persistent or significant disability/incapacity, or

• Constitutes a congenital anomaly or a birth defect; or

• Is medically significant, as determined by a qualified health professional, may jeopardize the subject, and may require medical or surgical intervention to prevent one of the outcomes listed above

• Major respiratory failure as per ICD-10

• Any adverse event causing the need for unblinding will be handled as a serious adverse event.

**Data Management**

**Documentation**

Case report forms (CRFs) will identify each subject by subject number (compilation of site number and randomization number) and subject's initials (per local regulatory guidelines). Originals or copies of all source documents, and correspondence will be kept on file at the investigational site.

The data required by the protocol will be recorded in the appropriate pages of CRFs. All source data will be available to the study monitor who may perform a 100% data check (comparison of the data recorded in the eCRF with those in the source documents). The source data will also be available for an audit by the Sponsor and all applicable regulatory agencies at any time.

**Data Collection**

In addition to the CRFs, individual subject files or subject medical records as well as Sponsor approved source data information will be maintained. These files constitute source data, and it is required that they be signed or initialed and dated by the staff recording the data. Data will be entered into CRFs. The CRFs will be kept current, so that they reflect the latest observations on the subjects enrolled in the study. The CRFs will include an audit trail to include changes made, reason for change, date of change, and person making changes.

The original signed informed consent form will be available for review at each study visit. The informed consent form will be in the appropriate section of the Investigator Regulatory Binder. All records will be kept in conformance to applicable national laws and regulations.

Analytical data from the local laboratory will be received and to file as appropriate along with source documents.

**Ethics & Good Clinical Practice**

This study will be carried out in compliance with the protocol and in accordance with all applicable standard operating procedures (SOPs). These will be designed to ensure adherence to GCP, as described in:

ICH Harmonized Tripartite Guidelines for Good Clinical Practice 1996. The Investigator agrees, when signing the protocol, to adhere to the instructions and procedures described in it, and thereby to adhere to the principles of GCP to which it conforms.

**Institutional Review Board/Independent Ethics Committee**

Before implementing this study, the protocol, the proposed informed consent form and other information to subjects will be reviewed by a properly constituted IRB/IEC. A signed and dated statement that the protocol and informed consent have been approved by the IRB/IEC will be given to the Sponsor before study initiation. The name and occupation of the chairman and the members of the IRB/IEC will be supplied to the Sponsor. This committee will approve any amendments to the protocol, other than administrative changes.

**Informed Consent**

Fully informed consent will be obtained before any study specific procedures are performed. The content and process of obtaining informed consent must be in accordance with all applicable ethical and regulatory requirements. Informed consent form must be signed by the subject voluntarily. In a situation where a subject is unable to provide consent for him/her, informed consent will be obtained from a Legally Authorized Representative (LAR) / next of kin / legally appointed individual in person in the presence of an impartial witness.

**Publication of Results**

Any formal presentation or publication of data from this study will require prior approval by the Sponsor, and may be considered as a joint publication by the Investigators and the Sponsor. Primary authorship may be determined by the Sponsor. Publication of subsets of data will be subject to review by the Sponsor. All data remain the property of the Sponsor, except, of course, copies maintained at each site in compliance with ICH GCP and other regulations.

**References**

- Hober D., Sane F., Jaidane H., Riedweg K., Goffard A., Desailloud R. Immunology in the clinic review series; focus on type 1 diabetes and viruses: Role of antibodies enhancing the infection with Coxsackievirus-B in the pathogenesis of type 1 diabetes. Clin Exp Immunol. 2012;168:47–51.
- Christou L. The global burden of bacterial and viral zoonotic infections. Clin Microbiol Infect. 2011;17:326–330.
- Sheu T.G., Deyde V.M., Okomo-Adhiambo M., Garten R.J., Xu X., Bright R.A. Surveillance for neuraminidase inhibitor resistance among human influenza A and B viruses circulating worldwide from 2004 to 2008. Antimicrob Agents Chemother. 2008;52:3284–3292.
- Locarnini S.A., Yuen L. Molecular genesis of drug-resistant and vaccine-escape HBV mutants. Antivir Ther. 2010;15:451–461.
- Geller C., Varbanov M., Duval R.E. Human coronaviruses: Insights into environmental resistance and its influence on the development of new antiseptic strategies. Viruses. 2012;4:3044–3068.
- Lin C.W., Tsai F.J., Tsai C.H., Lai C.C., Wan L., Ho T.Y. Anti-SARS coronavirus 3C-like protease effects of Isatis indigotica root and plant-derived phenolic compounds. Antivir Res. 2005;68:36–42.
- Cheng P.W., Chiang L.C., Yen M.H., Lin C.C. Bupleurum kaoi inhibits Coxsackie B virus type 1 infection of CCFS-1 cells by induction of type I interferons expression. Food Chem Toxicol. 2007;45:24–31.
- Back A.T., Lundkvist A. Dengue viruses-an overview. Infect Ecol Epidemiol. 2013;3:19839.
- Zandi K., Teoh B.T., Sam S.S., Wong P.F., Mustafa M.R., Abubakar S. Novel antiviral activity of baicalein against dengue virus. BMC Complement Altern Med. 2012;12:214.
- Low J.S., Wu K.X., Chen K.C., Ng M.M., Chu J.J. Narasin, a novel antiviral compound that blocks dengue virus protein expression. Antivir Ther. 2011;16:1203–1218.
- Wang S.M., Ho T.S., Lin H.C., Lei H.Y., Wang J.R., Liu C.C. Reemerging of enterovirus 71 in Taiwan: The age impact on disease severity. Eur J Clin Microbiol Infect Dis. 2012;31:1219–1224.
- Zhang L., Wang G., Hou W., Li P., Dulin A., Bonkovsky H.L. Contemporary clinical research of traditional Chinese medicines for chronic hepatitis B in China: An analytical review. Hepatology. 2010;51:690–698.
- Polyak S.J., Morishima C., Shuhart M.C., Wang C.C., Liu Y., Lee D.Y. Inhibition of T-cell inflammatory cytokines, hepatocyte NF-kappaB signaling, and HCV infection by standardized Silymarin. Gastroenterology. 2007;132:1925–1936.
- Cheng H.Y., Lin L.T., Huang H.H., Yang C.M., Lin C.C. Yin Chen Hao Tang, a Chinese prescription, inhibits both herpes simplex virus type-1 and type-2 infections in vitro. Antivir Res. 2008;77:14–19.
- Burton D.R., Desrosiers R.C., Doms R.W., Koff W.C., Kwong P.D., Moore J.P. HIV vaccine design and the neutralizing antibody problem. Nat Immunol. 2004;5:233–236.
- Pleschka S. Overview of influenza viruses. Curr Top Microbiol Immunol. 2013;370:1–20.
- Braciale T.J. Respiratory syncytial virus and T cells: Interplay between the virus and the host adaptive immune system. Proc Am Thorac Soc. 2005;2:141–146.
- Xie Y., Huang B., Yu K., Shi F., Liu T., Xu W. Caffeic acid derivatives: A new type of influenza neuraminidase inhibitors. Bioorg Med Chem Lett. 2013;23:3556–3560.
